# Comprehensive characterization of ferroptosis in hepatocellular carcinoma revealing the association with prognosis and tumor immune microenvironment

**DOI:** 10.3389/fonc.2023.1145380

**Published:** 2023-03-27

**Authors:** Jingjuan Zhu, Xiao Xu, Man Jiang, Fangfang Yang, Yingying Mei, Xiaochun Zhang

**Affiliations:** ^1^ Cancer Precision Medical Center, The Affiliated Hospital of Qingdao University, Qingdao University, Qingdao, China; ^2^ Qingdao Medical College, Qingdao University, Qingdao, China

**Keywords:** hepatocellular carcinoma, ferroptosis, molecular typing, prognosis, tumor microenvironment, immunotherapy

## Abstract

**Background:**

Ferroptosis is a type of regulatory cell death (RCD) mode that depends on iron-mediated oxidative damage. It has the potential to improve the efficacy of tumor immunotherapy by modulating the tumor microenvironment (TME). Currently, immunotherapy has significantly improved the overall treatment strategy for advanced hepatocellular carcinoma (HCC), but the distinct immune microenvironment and high tolerance to the immune make massive differences in the immunotherapy effect of HCC patients. As a result, it is imperative to classify HCC patients who may benefit from immune checkpoint therapy. Simultaneously, the predictive value of ferroptosis in HCC and its potential role in TME immune cell infiltration also need to be further clarified.

**Methods:**

Three ferroptosis molecular models were built on the basis of mRNA expression profiles of ferroptosis-related genes (FRGs), with notable variations in immunocyte infiltration, biological function, and survival prediction. In order to further investigate the predictive impact of immunotherapy response in HCC patients, the ferroptosis score was constructed using the principal component analysis (PCA) algorithm to quantify the ferroptosis molecular models of individual tumors.

**Results:**

In HCC, there were three totally different ferroptosis molecular models. The ferroptosis score can be used to assess genetic variation, immunotherapy response, TME characteristics, and prognosis. Notably, tumors with low ferroptosis scores have extensive tumor mutations and immune exhaustion, which are associated with a poor prognosis and enhanced immunotherapy response.

**Conclusions:**

Our study indicates that ferroptosis plays an indispensable role in the regulation of the tumor immune microenvironment. For HCC, the ferroptosis score is an independent prognostic indicator. Assessing the molecular model of ferroptosis in individual tumors will assist us in better understanding the characteristics of TME, predicting the effect of immunotherapy in HCC patients, and thus guiding a more reasonable immunotherapy program.

## 1 Introduction

Hepatic cancer, especially hepatocellular carcinoma (HCC), which accounts for more than 90% of primary hepatic tumors, is the third leading cause of tumor-related deaths worldwide (1). In recent years, immunotherapy such as immune checkpoint inhibitors (ICIs) has completely replaced systematic chemotherapy as the first-line treatment method for advanced HCC (2, 3). The adoption of the Atezolizumab-Bevacizumab protocol as standard treatment, in particular, heralds the beginning of a revolutionary age (4). However, the complex pathophysiology, distinctive heterogeneity, and high immunological tolerance of HCC contribute significantly to variation in the therapeutic impact of immunotherapy in patients (5, 6). Numerous mechanisms, including immune evasion, dysfunction of effector T lymphocytes, immunosuppression, and poor tumor antigen expression, are present in the microenvironment of HCC (6). Any of these potential processes could be a formidable impediment to immunotherapy. The occurrence and development of HCC are thought to be a multi-step process, and the precise molecular processes leading to the formation of HCC have traditionally been the focus of HCC research. In the past, many researchers have discussed it from different perspectives. For instance, the cholangiocarcinoma-like (CCL) signature (7), the hepatoblastoma 16 gene (HB16) signature (8), the NCI proliferation (NCIP) signature (9), the hepatic stem cells (HS) signature (10), the 65 genes recurrence risk score (RS65 score) (11), the Seoul National University recurrence (SNUR) signature (12), the Hippo pathway signature (13), and the Hoshida signature (14). These molecular typing based on multi-omic data elaborated the genetic and immunological characteristics of HCC patients from different perspectives, which is the cornerstone for directing accurate treatment. Therefore, it is essential to perform molecular classification of HCC patients who may benefit from immune checkpoint therapy.

Ferroptosis is distinct from cell necrosis, apoptosis, and autophagy (15, 16). Iron metabolism disruption and reactive oxygen species (ROS) buildup resulting in lipid peroxidation are the main factors contributing to ferroptosis (17). Induction of ferroptosis has emerged as a promising cancer treatment option in recent years, especially for refractory malignant tumors (18, 19). Ferroptosis-related lipid peroxides encourage dendritic cells to identify, phagocytose, and handle tumor antigens before presenting them to CD8+T lymphocytes as a recognition signal. CD8+T cells release IFN-γ, which inhibits the cystine absorption of tumor cells and activates cytotoxic T lymphocytes, hence enhancing tumor immunotherapy (20–22). These findings suggest that ferroptosis has a profound impact on TME and immunotherapy. Ferroptosis provides an innovative idea for the development of new candidate drugs for the treatment of refractory cancers. After acquired resistance to EGFR-TKIs, EGFR-mutated lung cancer cells showed increased sensitivity to ferroptosis inducers (23). Jiang et al. reported that TYRO3 can promote the development of the tumor microenvironment by reducing the ratio of M1/M2 macrophages while inhibiting TYRO3 can promote tumor ferroptosis and make drug-resistant tumors sensitive to PD-1 therapy (24). A recent study found that the small molecule MMRi62 can induce ferroptosis in pancreatic ductal adenocarcinoma (PDAC) cells carrying KRAS and/or p53 gene mutations, thus inhibiting tumor growth and preventing metastasis (25). These recent studies indicate that the induction of ferroptosis may overcome the drug resistance of targeting and immunotherapy. When transforming ferroptosis into clinical application, it will be particularly important to develop specific therapies that can induce ferroptosis in cancer cells while avoiding systemic adverse reactions. In this regard, nanoparticle ferroptosis inducers provide unique advantages (26). In 2021, the scientific research team led by Jianlin Shi proposed a non-ferrous ferroptosis-like strategy based on a hybrid CoMoO4-phosphomolybdic acid nanosheet (CPMNS). The ferroptosis-like cell death process is triggered by increasing ROS, depleting GSH (glutathione), and regulating GPX4 activity. Both *in vitro* and *in vivo* results have proved significant anticancer efficacy, indicating that this ferroptosis-like death strategy supported by CPMNS extends the applicability of the concept of ferroptosis to the process of ferroptosis-like death induced by non-ferrous metals, which will contribute to future progress in the field of cancer treatment programs (27). Last but not least, we currently lack biomarkers to mark ferroptosis in the body. The exploration of suitable biomarkers will facilitate the development of further *in vivo* research and clinical surveillance (28, 29).

The inflammatory state of TME has been proven to be essential for the occurrence, development, invasion, and metastasis of almost all solid tumors (30). In most cases, HCC is the result of chronic liver inflammation that leads to the formation of a complex TME composed of immune cells and stromal cells. TME involves the development of metastasis and drug resistance. This has become a challenge in the treatment of HCC patients because it influences the response to targeted and immunotherapy (31). In the past decade, immunotherapy has developed rapidly and has been recognized as a key strategy for controlling the progression of malignant tumors. PD-1/PD-L1 inhibitors have been approved for many solid tumors and hematological malignancies, including non-small cell lung cancer, melanoma, urothelial carcinoma, esophageal carcinoma, renal cell carcinoma, and Hodgkin’s lymphoma (32). According to the results of the IMbrave150 study, Atezolizumab combined with bevacizumab has been approved for the first-line treatment of unresectable locally advanced or metastatic hepatocellular carcinoma (33). Another promising immune checkpoint inhibitor treatment strategy is the combination of Durvalumab (PD-L1 inhibitor) and Tremelimumab (CTLA-4 inhibitor). PD-L1 and CTLA-4 are both inhibitory molecules expressed in T cells. Treatment with these two antibodies recently showed promising results in the phase III HIMALAYA clinical trial (NCT03298451). Their effectiveness in improving the survival of HCC patients highlights the role of T cells in the treatment of HCC (34). Chimeric antigen receptor T cell (CAR-T) therapy is an innovative type of tumor immunotherapy. Through genetic engineering technology, T cells can specifically recognize tumor-related antigens, thus exerting anti-tumor effects (35). To date, five CAR-T cell therapies have been approved for hematological malignancies. Several CAR-T therapies are currently undergoing clinical trials for HCC targeting a variety of surface and intracellular antigens (36). It is noteworthy that the characteristics of hypoxia and nutrient deprivation of TME have seriously weakened the adaptability and efficacy of CAR-T cells, emphasizing the need for more complex engineering strategies (36). Another new option for HCC immunotherapy is adaptive T cell transfer of gamma-delta T cells ( γδ T cells). Low infiltration of γδ T cells in peritumoral liver tissue is associated with a higher recurrence rate of HCC and predicts postoperative recurrence (37). Adoptive transfer of allogeneic-γδ T cells in combination with local interventional therapy has an encouraging clinical effect against HCC and intrahepatic cholangiocarcinoma (ICC) (38). At present, many drugs targeting the tumor microenvironment are under development, including synthetic drugs, biotherapeutics, and vaccines. Personalized treatment regimens will be needed to achieve maximum clinical benefits for patients.

Epithelial-mesenchymal transformation (EMT) in TME was originally thought to primarily be associated with invasive metastasis of cancer cells, but new research has revealed that EMT is an important mechanism of tumor treatment resistance (39–41). Previous research has demonstrated that tumor microenvironment (such as hypoxia), numerous growth factors, and carcinogenic-associated signaling pathways (such as TGF-β, Notch, MAPK, and KRAS signaling pathways), can activate the EMT process (42–44). Mariathasan et al. gathered a set of EMT marker genes, including EMT1 (breast cancer) (45), EMT2 (urothelial carcinoma) (46), EMT3 (metastatic melanoma) (47), angiogenesis indicators (48), and WNT targets (49). They studied a large number of patients with urothelial carcinoma who were taking an anti-PD-L1 medication and discovered that a favorable immune response was connected to CD8+T effector cell phenotype and tumor mutation burden (TMB) (50). Schreiber et al. found that inhibition of glutathione peroxidase 4 (GPX4) induced ferroptosis in mesenchymal resistant cancer cells (51). Similar to GPX4-dependent mesenchymal resistant cancer cells, persistently drug-resistant cancer cells are also highly sensitive to ferroptosis (52). As a result, further understanding the role of ferroptosis in the tumor microenvironment and EMT regulation would aid in the investigation of tumor drug resistance mechanisms.

In this study, we screened three hub genes and performed pan-cancer analysis. The expression verification and survival analysis were carried out in the validation queue of our hospital. We structured three ferroptosis molecular patterns and found that their prognosis and TME characteristics were significantly different. Then we identified the ferroptosis scoring system, which can accurately predict the effect of immunotherapy, suggesting that ferroptosis has a significant impact on the treatment of advanced HCC.

## 2 Material and methods

### 2.1 Data collection

This study analyzed mRNA expression data and clinical information of 371 members in the TCGA-LIHC cohort available in The Cancer Genome Atlas (TCGA, https://portal.gdc.cancer.gov/repository) database. Additionally, 167 samples from the GSE76427 cohort were obtained from the Gene Expression Omnibus (GEO) database (https://www.ncbi.nlm.nih.gov/geo). To further validate the results, we analyzed the mRNA expression data of 240 samples from the International Cancer Genome Consortium (ICGC, https://dcc.icgc.org/) database.

The TCGA-LIHC copy number variation (CNV) information is derived from the UCSC Xena database (https://xena.ucsc.edu/). The TCGA-LIHC clinical information is derived from the UCSC Xena database and research published by the TCGA team in *Cell (* 53). Afterward, FRGs were obtained from FerrDb, which consists of a database of ferroptosis regulators, markers, and associations between ferroptosis and various diseases (54). After removing duplicate genes, in all, 258 FRGs were available for analysis (**Supplementary Table 1**). In addition, the data contained in TCGA, GEO, ICGC, and FerrDb is publicly available. TCGA, GEO, and ICGC policies and guidelines for data acquisition and publication were strictly followed in the conduct of this study.

### 2.2 FRGs screening and protein-protein interaction network construction

The RNA high-throughput sequencing data in FPKM form was converted to TPM using the “TCGAbiolinks” (version, 2.26.0) R package (55). The “limma” (version, 3.54.0) package was used to analyze 373 HCC samples and 49 paracancerous tissues from the TCGA-LIHC cohort (56). Thus, differentially expressed FRGs were identified (FDR<0.01, |logFC|>1). Univariate Cox regression analysis was performed among FRGs, and *p*<0.01 was used as a screening condition to identify potential prognostic genes affecting overall survival (OS). Based on these FRGs, the PPI between proteins were generated by the STRING database. Following this, hub genes were identified *via* Cytoscape (version, 3.9.0). The confidence score was set as a score<0.4.

### 2.3 Pan-cancer analysis

To analyze the differential expression and survival prediction of hub genes in 33 cancers, we collected gene expression information and relevant clinical data from the TCGA database for 33 tumor types.

### 2.4 Immunohistochemical analysis of clinical validation cohort

We obtained 69 surgical specimens of hepatocellular carcinoma and 41 matched paracancerous tissues from the Affiliated Hospital of Qingdao University (hereinafter referred to as our hospital), as well as the corresponding clinical information. To assess the expression levels of hub genes (HRAS, SLC7A11, and SLC2A1), immunohistochemistry (IHC) was accomplished by GTVisionTM III Detection Systems (Genetech, Shanghai, China) and antibodies (18295-1-AP, ab115730, ab37185) according to the manufacturer’s instructions. The immunohistochemical staining was assessed by two pathologists who were uninformed of the clinical information. When their assessments differ, the third pathologist will undertake an independent examination. For each pathological section, we observed ten optical fields under a high-power lens (× 400). We took the IHC staining score as the final staining judged criteria. IHC staining score = staining area score × staining intensity score. The staining area score was estimated on a scale of 0-4 (0, ≤10%; 1, 11-25%; 2, 26-50%; 3, 51-75%; 4, ≥75%); the staining intensity score was classified as 0 (negative), 1 (weak), 2 (moderate), or 3 (strong). We grouped the IHC staining score to demonstrate the relationship between hub gene expression and patient survival. The IHC staining score below six was defined as low expression group, while the score over six was defined as high expression group. Moreover, the differences in IHC staining scores of hub genes between tumors and adjacent normal tissues were performed using the Wilcoxon rank sum test.

### 2.5 Identification of the ferroptosis molecular patterns

We recognized the ferroptosis molecular patterns on the basis of FRGs mRNA expression profiles by using the “ConsensuClusterPlus” package (57). The patients from the TCGA-LIHC and GSE 76427 were then classified for further investigation. The consensus clustering algorithm calculated the number and stability of clusters.

### 2.6 Enrichment of functional properties and TME immune infiltration in ferroptosis

In order to search for potential biological behaviors between ferroptosis molecular patterns, the GO and KEGG functional analyses were performed by “clusterProfiler” (version, 4.6.0) package (FDR<0.05).

From the MSigDB database, we obtained the gene set “c2.cp.kegg.v7.4.symbols” for our GSVA analysis by “GSVA” (version, 1.46.0) packages (FDR<0.05). In general, GSVA is an unsupervised, nonparametric approach for estimating the levels of variation within biological pathways and processes in expression datasets.

The mechanism of TME features generation was then investigated using a Single Sample Gene Set Enrichment Analysis (ssGSEA) (58). According to the expression of a set of tumor-infiltrating immune cells (TIICs) and immune function marker genes obtained from Bindea et al., the TIICs enrichment score and immune function of each HCC sample were quantitatively assessed. Mariathasan et al. identified and characterized a series of gene sets that relate to the following biological processes: antigen processing machinery; epithelial-mesenchymal transition (EMT) markers consisting of EMT1, EMT2, and EMT3; angiogenesis signature; Pan-fibroblast TGF-β response signature (Pan-FTBRS); WNT targets; FGFR3 related genes (**Supplementary Table 2**) (50). We retrieved associated gene sets from the MSigDB database, to further illuminate the processes by which ferroptosis influences the tumor immune microenvironment including the following: TGF-EMT down-regulation signal pathways; TGF-EMT up-regulation signal pathways; MAPK signal pathways; NOTCH signal pathways; KRAS up-regulation signal pathways; KRAS down-regulation signal pathways; hallmark-hypoxia; HIF-1 signal pathways to increase oxygen delivery; HIF-1 signal pathways to decrease oxygen consumption.

### 2.7 The ferroptosis score

We identified differentially expressed genes (DEGs) associated with the ferroptosis pattern through the “limma” package and screened prognostic genes using Univariate Cox regression models (*p*<0.05). We used principal component analysis (PCA) to quantify the ferroptosis molecular models of individual tumors and constructed a scoring system, which was termed the ferroptosis score. We defined the ferroptosis score as follows: Ferroptosis score =∑ (PC1i + PC2i), where i denotes the expression of prognostic DEGs associated with ferroptosis molecular models (59, 60). Patients were divided into low and high ferroptosis score groups in accordance with the threshold of -23.27889 established by “Survminer”.

The independent prognostic value of the ferroptosis score was determined with Univariate and Multivariate Cox analysis. Next, a prediction model was constructed by integrating the ferroptosis score and other independent clinical risk factors according to the prognostic multivariate profile. A nomogram plot was used to visualize the relationship between the variables in the prediction model by following a certain scale in the same plane. A prognostic calibration plot was used for fit analysis of the model to the actual situation and to determine predictive efficacy.

### 2.8 Assessment of tumor mutation burden and immunotherapy response

Based on the MAF files, somatic mutation data was visualized using the “maftools” (version, 2.14.0) package (61). We calculated the TMB of each patient as follows: TMB= (total count of variants)/(total length of exons).

In addition, Jiang et al. proposed TIDE method in order to simulate immune escape mechanisms in cancer, including T cell dysfunction and T cell rejection (62). In this study, TIDE was used to assess response to immunotherapy. A higher TIDE score not only indicates that the tumor has an immune avoidance phenotype, but also predicts a poor response to ICIs in cancer patients.

### 2.9 Chemotherapeutic drug sensitivity prediction

The sensitivity of ferroptosis to chemotherapeutic agents was assessed by the Genomics of Drug Sensitivity in Cancer (GDSC; https://www.cancerrxgene.org/) database (63). The half maximum inhibitory concentration (IC_50_) was calculated by the “pRRophetic” (64).

### 2.10 Statistical analysis

We used Wilcoxon rank-sum tests for comparing the two groups and Kruskal-Wallis tests for assessing multiple comparisons. Based on the output from the “survminer” package, a dividing point was determined for each subgroup. In order to analyze the survival times of different subgroups, Kaplan-Meier curves and log-rank tests were used.

## 3 Results

### 3.1 The landscape of genetic variation of FRGs in hepatic cancer

**Figure 1:**
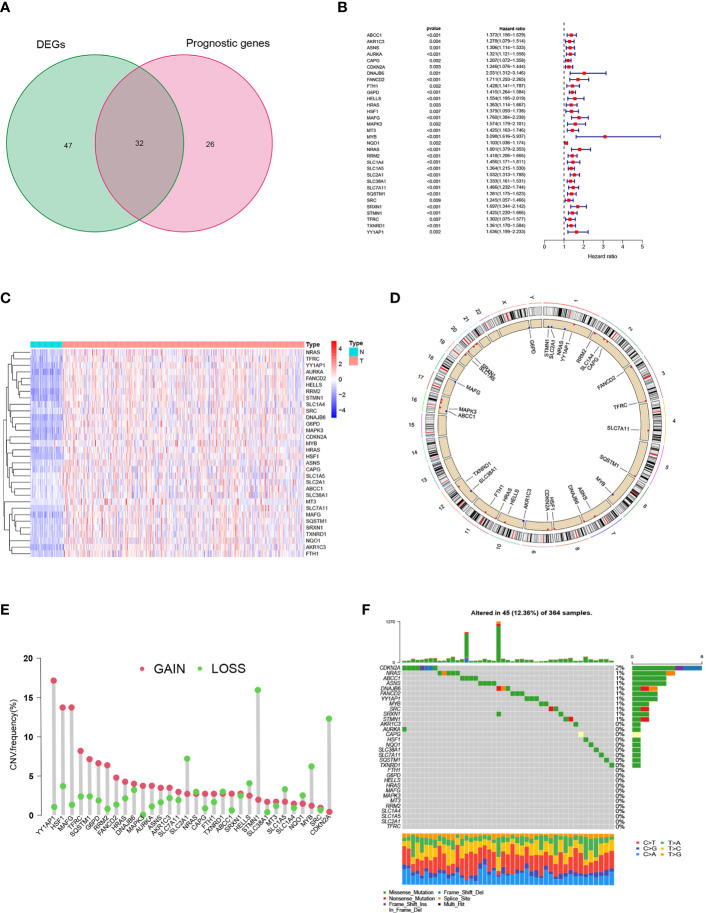
Prognostic ferroptosis-related gene (FRGs) differentially expressed in TCGA. **(A)** Venn graph showing the intersection of prognostic genes and differentially expressed genes. **(B)** Forest plots illustrating the Univariate Cox regression analysis of overlapping genes. **(C)** Expression of overlapping genes in tumor tissue. **(D)** CNV alteration locations for 32 FRGs. **(E)** The frequency of CNV variation of 32 FRGs. Alteration frequency was represented by the height of the column. Green dots indicating deletions; red dots indicating amplifications. **(F)** The mutation frequency of 32 FRGs in 364 patients with HCC. One patient was represented by each column. The upper bar plot indicated the extent of tumor mutations. Numbers on the right indicated the frequency of mutations in each gene. The right bar plot showed the proportions of the different types of variants. Stacked bar plot of each sample was able to show the fraction of conversions.

A total of 258 ferroptosis-related genes (FRGs) were included in the analysis. We found that in the TCGA-LIHC cohort, 30.6% of FRGs (79/258), was differentially expressed in HCC tissues and non-tumor para-cancer tissues (FDR<0.01, |logFC|>1; **Supplementary Table 3**). As a result of a subsequent Univariate Cox regression analysis, 58 FRGs were correlated with overall survival (OS) (*p*<0.01; **Supplementary Table 4**). By cross-overlapping 79 differentially expressed FRGs and prognostic related genes, we obtained 32 differentially expressed prognostic related FRGs (**Figure 1A**). The forest map displayed the Hazard ratios of the 32 FRGs in the single-factor Cox regression analysis (**Figure 1B**). The heat map showed that the expression levels of 32 FRGs were significantly different between tumor tissues and normal tissues. FRGs were significantly enriched in tumor tissues (**Figure 1C**). We also examined the incidence of somatic mutations and copy number variations (CNVs) for FRGs. According to the position of the 32 FRGs on the chromosome, the CNV changes are shown in **Figure 1D**. As a result of the CNV variation frequency analysis, CNV variation was very common in FRGs, most of which focused on copy number amplification (**Figure 1E**). We found that CDKN2A had the highest frequency of mutation in HCC samples, followed by NARS (**Figure 1F**). The above analysis presented that the expression of FRGs in normal liver tissue and HCC tissue was highly heterogeneous, suggesting that the modifications in the expression of FRGs may contribute significantly to the occurrence and development of hepatocellular carcinoma.

### 3.2 Hub gene screening and Pan-cancer analysis

**Figure 2:**
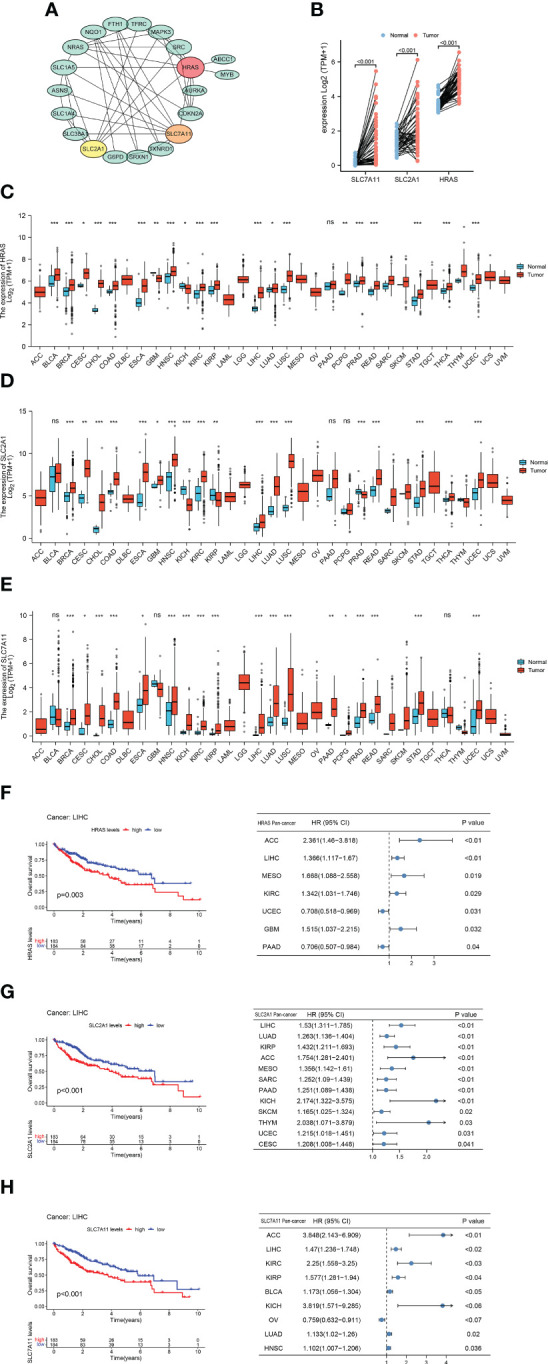
Hub Gene Screening and Pan-carcinoma Analysis. **(A)** Identified PPI hub genes. **(B)** Differential expression of HRAS, SLC2A1, and SLC7A11 in HCC paired samples. **(C)** Differential expression of HRAS in 33 cancers. ns, not significant; *p<0.05; **p<0.01; ***p<0.001. **(D)** Differential expression of SLC2A1 in 33 cancers. ns: not significant; *p<0.05; **p<0.01; ***p<0.001. **(E)** Differential expression of SLC7A11 in 33 cancers. ns: not significant; *p<0.05; **p<0.01; ***p<0.001. **(F)** Kaplan-Meier survival curve of HRAS and pan-carcinoma Univariate Cox regression analysis. **(G)** Kaplan-Meier survival curve of SLC2A1 and pan-carcinoma Univariate Cox regression analysis. **(H)** Kaplan-Meier survival curve of SLC7A11 and pan-carcinoma Univariate Cox regression analysis.

We generated a protein-protein interaction network (PPI) for FRGs and identified three hub genes *via* Cytoscape, including HARS, SLC7A11, and SLC2A1 (**Figure 2A**, **Supplementary Figure 1A**). These three FRGs showed significant differential expression in matched samples of cancer and Normal paracancerous tissue in TCGA-LIHC cohort (**Figure 2B**). Pan-cancer analysis demonstrated that these three FRGs were significant differential expression in most cancers (**Figures 2C–E**). The survival analysis of these three genes also showed their potential role in survival prediction (**Figures 2F–H**).

### 3.3 Clinical cohort verification

**Figure 3:**
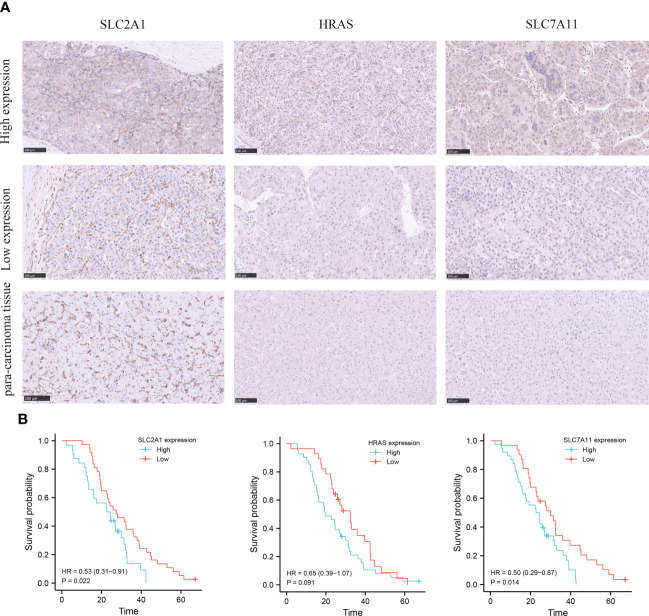
Immunohistochemical (IHC) analysis of clinical validation cohort. **(A)** Comparison of SLC2A1, HRAS, and SLC7A11 expression in HCC tissues and adjacent tissues. **(B)** Survival curves of HCC patients with high and low SLC2A1, HRAS, and SLC7A11 expression.

In order to evaluate the expression level of hub genes (HRAS, SLC7A11 and SLC2A1) in hepatocellular carcinoma, we conducted immunohistochemical (IHC) analysis. The expression of HRAS, SLC2A1, and SLC7A11 was positive in the majority of specimens from the validation cohort in our hospital. Among them, SLC7A11 has strongly stained in 38 (55.1%) specimens, HRAS was strongly stained in 41 (59.4%) specimens, and SLC2A1 was strongly stained in 32 (46.4%) specimens (**Figure 3A**; **Supplementary Table 5**). Finally, the Kaplan-Meier curve showed that patients with high gene expression had a shorter survival than patients with low gene expression (**Figure 3B**).

### 3.4 Ferroptosis molecular patterns with different TME features and function

**Figure 4:**
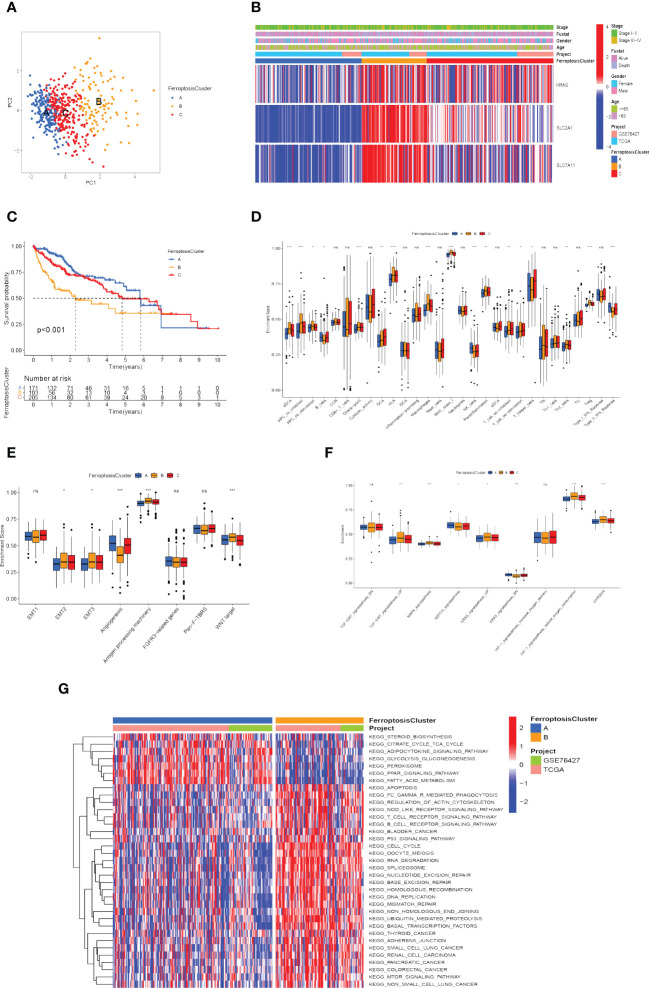
Recognition of ferroptosis molecular patterns with specific TME features and functional enrichment analysis. **(A)** Principal component analysis of hub gene expression profiles distinguished three ferroptosis clusters, A (blue), B (yellow), and C (red). **(B)** Heat map displaying the correlation between the hub genes expression and ferroptosis clusters. **(C)** Kaplan-Meier curves for the three molecular patterns of HCC patients. **(D)** Box plots displayed the levels of immune infiltration in the three patterns. ns, not significant; *p<0.05; **p<0.01; ***p<0.001. **(E, F)** Differences in stromal activation pathway **(E)** and carcinogenesis-related pathways **(F)** in the three ferroptosis clusters. ns, not significant; *p<0.05; **p<0.01; ***p<0.001. **(G)** GSVA analysis revealed distinct activations of biological pathways in ferroptosis clusters. Blue represented the inhibition pathway and red represented the activation pathway.

According to the expression of the three FRGs in the TCGA-LIHC and GSE76427 cohorts, HCC patients were subdivided into three molecular patterns by unsupervised cluster analysis, termed ferroptosis clusters A, B, and C (A: *n*=194, B: *n*=107, C: *n*=236; **Supplementary Figure 1B**). It was demonstrated by the principal component analysis (PCA) that the three subtypes were entirely separate (**Figure 4A**). Among the three molecular patterns, the three FRGs were significantly highly expressed in cluster B, and appreciably low expressed in cluster A (**Figure 4B**). Prognostic analysis revealed an exceedingly favorable outcome in ferroptosis cluster A, whereas cluster B had the most detrimental prognosis (*p*<0.001; **Figure 4C**).

Afterward, we evaluated the correlation among these patterns and TME features. Immune cell infiltration varied greatly between the three molecular patterns, especially for ferroptosis cluster A, which was remarkably rich in NK cells and type-II IFN (IFN-γ) response. Ferroptosis cluster B was abounding in activated dendritic cells (aDCs), antigen-presenting cells (APCs), check-point, human leukocyte antigen (HLA), macrophages, regulatory T cells (Tregs), major histocompatibility complex (MHC) class I (**Figure 4D**). We also explored the relationship between ferroptosis clusters and various biological processes. The results manifested that EMT2, EMT3, antigen processing machinery, and WNT targets scored the highest in ferroptosis cluster B, as well as angiogenesis signature, which was significantly enriched in ferroptosis cluster A (**Figure 4E**). In particular, ferroptosis cluster B was found to be significantly enriched in hypoxia-related signaling pathways and EMT-related signaling pathways (such as TGF-β, MAPK, and KRAS signaling pathway) (**Figure 4F**). We further assessed the hypoxia status of the three ferroptosis molecular patterns using the Buffa Hypoxia Score and found equally significant differences (**Supplementary Figure 1C**) (65). Hypoxia and EMT are two important tumor microenvironmental biological processes that significantly affect the prognosis of HCC patients, which makes it necessary to pay attention to their relationship with ferroptosis. Their interaction may be an important clue to observing the effect of ferroptosis on immunotherapy and the prognosis of hepatocellular carcinoma.

Subsequently, we explored the differences in ferroptosis molecular patterns in biological signaling pathways. As shown in **Figure 4G**, ferroptosis cluster A was markedly enriched in fatty acid metabolism, adipocytokine signaling pathway, glycolysis and gluconeogenesis, and PPAR signaling pathways. The ferroptosis cluster B presented enrichment pathways prominently related to the p53 signaling pathway, T cell receptor signaling pathway, adherens junction, mTOR signaling pathway, Oocyte meiosis, ubiquitin-mediated proteolysis, cell cycle, and DNA damage repair. Therefore, it is reasonable to speculate that cluster B may be associated with invasive HCC, while cluster A may be associated with metabolic disorders.

### 3.5 Generation of ferroptosis-related genomic patterns

**Figure 5:**
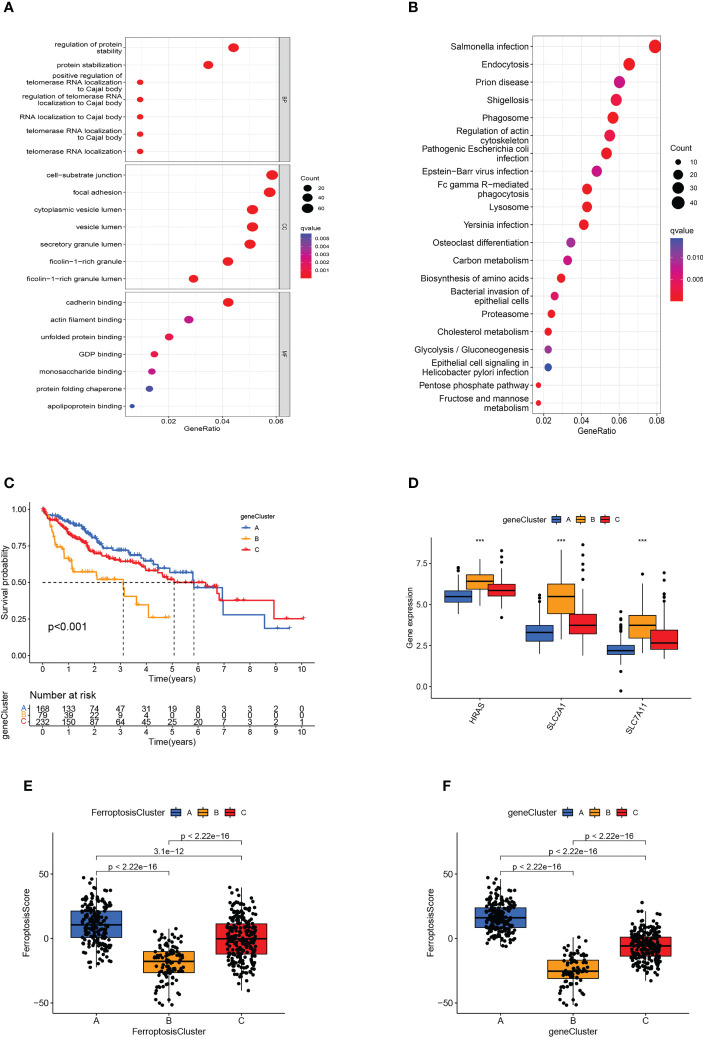
Generation of ferroptosis-related genomic patterns and the ferroptosis score. **(A, B)** GO **(A)** and KEGG **(B)** analysis based on differentially expressed genes. **(C)** Kaplan-Meier curves for the three gene clusters of HCC patients. **(D)** Box plots showed hub gene expression in the three gene clusters. ***p<0.001. **(E, F)** Box plots displayed the differences in ferroptosis scores among the three ferroptosis clusters **(E)** and the three gene clusters **(F)**.

As a means of further detecting potential biological processes among the ferroptosis molecular patterns, we determined 1139 DEGs related to ferroptosis molecular patterns, then performed GO and KEGG enrichment analysis (**Figures 5A, B**). As expected, DEGs were enriched in a number of molecular functions related to cell-substrate junction, focal adhesion, ficolin-1-rich granule, regulation of telomerase RNA localization to Cajal body, Fc epsilon RI signaling pathway, Cholesterol metabolism, which confirmed again that ferroptosis molecular patterns played an effective role in tumor immune activation, invasion and proliferation, and metabolic disorder.

In order to further verify the regulatory mechanism, the Univariate Cox analysis was conducted on these DEGs and screened out 794 prognostic DEGs (*p*<0.05). On the basis of these prognostic DEGs, we conducted an unsupervised cluster analysis, the TCGA-LIHC and GSE76427 patients were classified into three ferroptosis genomic patterns and we named them gene clusters A, B, and C (A: *n*=214, B: *n*=84, and C: *n*=239; **Supplementary Figure 1D**). Further investigation was carried out on the prognostic implications of ferroptosis gene clusters. In general, it was found that subjects in gene cluster A recorded a longer OS, whereas those in gene cluster B exhibited a more pessimistic outlook (*p*<0.001; **Figure 5C**). The expression of HRAS, SLC2A1, and SLC7A11 differed significantly between the three gene clusters, which also matched the expected outcomes of the ferroptosis molecular patterns (**Figure 5D**). The heatmap showed that ferroptosis-related genomic patterns were almost identical to the ferroptosis molecular patterns (**Supplementary Figure 1E**).

### 3.6 Construction of ferroptosis scoring system

However, our previous studies were based on patient populations. Considering individual heterogeneity and the complex mechanisms of HCC, we developed a PCA-based scoring algorithm to quantify the ferroptosis molecular pattern in individual patients, which we call the ferroptosis score. The ferroptosis scores in the ferroptosis clusters, as well as the gene clusters, were substantially different (*p*<0.01; **Figures 5E, F**). Ferroptosis cluster B patients had the poorest prognosis and the lowest ferroptosis score, as predicted, whereas cluster A patients had the opposite features (**Figure 5E**). The gene cluster produced the expected result in the ferroptosis score (**Figure 5F**).

### 3.7 Development of an independent prognostic model for HCC based on ferroptosis

**Figure 6:**
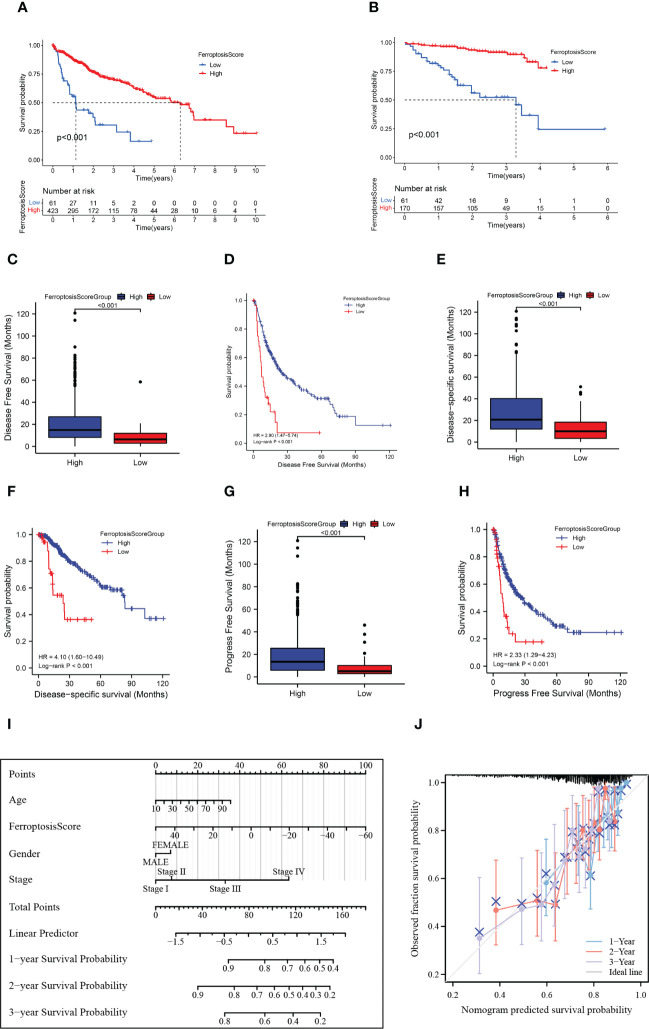
HCC prognosis based on the ferroptosis score. **(A, B)** Kaplan-Meier survival curves for the ferroptosis score groups in TCGA+GEO **(A)** and ICGC **(B)**. **(C)** Box plots showed the differences in disease-free survival (DFS) between ferroptosis score groups. **(D)** Kaplan-Meier curves for the ferroptosis score groups in disease-free survival (DFS). **(E)** Box plots showed the differences in disease special survival (DSS) between ferroptosis score groups. **(F)** Kaplan-Meier curves for the ferroptosis score groups in disease special survival (DSS). **(G)** Box plots showed the differences in progression-free survival (PFS) between ferroptosis score groups. **(H)** Kaplan-Meier curves for the ferroptosis score groups in progression-free survival (PFS). **(I)** Nomogram plot of prognostic multivariate regression model. **(J)** Prognostic Calibration plot evaluating the fit analysis of the model to the actual situation.

We investigated the significance of the ferroptosis score in forecasting survival. Afterward, they were categorized into two groups: high ferroptosis and low ferroptosis (high group: *n*=423, low group: *n*=61). Consistent with our previous research, TCGA and GEO samples with low ferroptosis scores implied a more adverse prognosis than those with high ferroptosis scores (*p*<0.001; **Figure 6A**). We also used the ICGC cohort for validation and obtained consistent results (*p*<0.001; **Figure 6B**). Ferroptosis score also showed good predictive power in other indicators of clinical benefit, for example, disease special survival (DSS), disease-free survival (DFS), and progression-free survival (PFS) (*p*<0.001; **Figures 6C–H**). In the univariate and multivariate cox regression analysis, the ferroptosis score showed significantly superior survival prediction ability compared with other molecular classifications in previous studies (**Supplementary Figure 1F**).

We further established a nomogram plot to verify the accuracy of the prediction of the ferroptosis score in HCC (**Figure 6I**). We assigned a risk score to each clinical risk variable, including stage, age, gender, and ferroptosis score. Compared with other clinical features, the highest number of risk points was contributed by the ferroptosis score (from -60 to 50). The calibration curve of 1-, 2-, and 3-year OS was illustrated in **Figure 6J**. When compared to the actual situation, the predicted 1-year survival rate, 3-year survival rate, and 5-year survival rate of the model are close to the diagonal, indicating that the ferroptosis scoring model has a strong fitting effect.

### 3.8 Correlation between ferroptosis score and clinical features

We explored the relationship between clinical characteristics, molecular characteristics in previous studies and ferroptosis score group (**Supplementary Table 6**). Patients in the high score group have clinical features related to good prognosis (such as relatively low Child-Pugh classification, Stage, Grade, AFP). The median BMI of patients in the low ferroptosis score group was 22.80, while that in the high ferroptosis score group was 24.98. The Wilcoxon rank sum test showed statistically significant differences between the two groups (*p*=0.011).

We discovered that low score group patients had significant aggressive features and embryonic stem cell-like expression traits (ES signature), including CCL subtype (CCL feature), HB16 cluster 2, SOH subtype (HIPPO), HS subtype (NCIHS), high RS65 score, and NCIP cluster A (**Supplementary Table 6**). This indirectly confirmed that the ferroptosis molecular patterns may represent different developmental stages of HCC origin cells or different transformation mechanisms.

### 3.9 TMB characteristic of ferroptosis score

**Figure 7:**
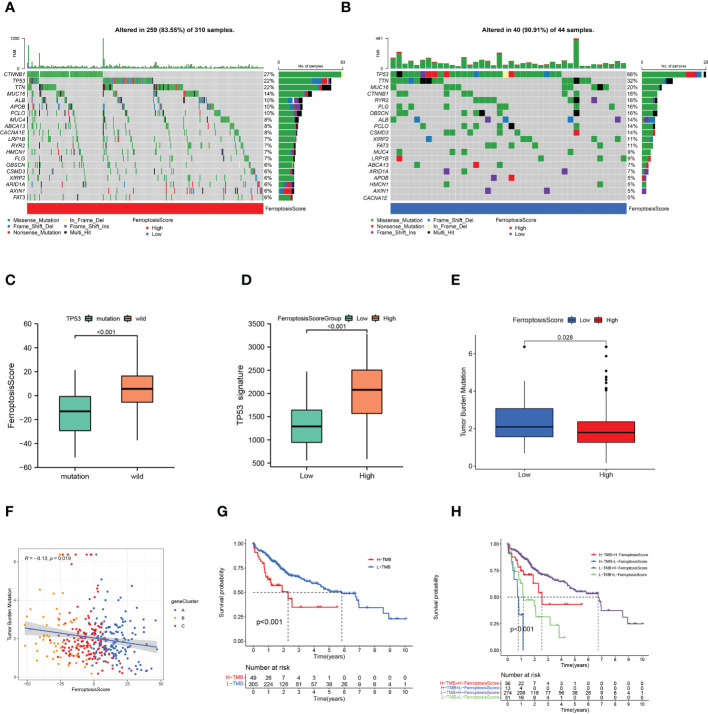
Ferroptosis score and tumor mutation burden. **(A, B)** Tumor somatic mutation waterfall plots established for those with high ferroptosis scores **(A)** and low ferroptosis scores **(B)**. **(C)** Box plot illustrated the difference of ferroptosis score between the TP53 mutation status. **(D)** Box plot illustrated the difference of p53 signature between the ferroptosis score groups. **(E)** Box plot illustrated the differences between the ferroptosis score groups in tumor mutation burden. **(F)** Relationships among ferroptosis score, tumor mutation burden, and gene clusters. **(G)** Kaplan-Meier curves of high and low tumor mutation burden patients. **(H)** Kaplan-Meier curves based on both the ferroptosis score and tumor mutation burden.

The waterfall plots showed the 20 genes with the highest mutation frequency in the somatic mutation data of patients in the TCGA-LIHC cohort. We compared the differences in mutation landscape between the two ferroptosis score groups. **Figures 7A, B** demonstrated that patients with low ferroptosis scores suffered from a greater tumor mutation burden than patients with high ferroptosis scores. It should be noted that the mutation frequency of TP53 in the low ferroptosis score group was significantly increased, and the ferroptosis score of TP53 mutant samples was also significantly lower than that of wild-type samples (*p*<0.01; **Figure 7C**). The association of TP53 mutations with poor prognosis is well known in many cancer types. In order to more accurately evaluate p53 functional status, the TCGA team developed a p53 signature (53). HCC patients with low p53 expression displayed a significantly reduced OS relative to their high p53 signature counterparts. We found that higher ferroptosis scores also had significantly elevated p53 signatures (**Figure 7D**).

The quantification analysis of TMB confirmed that low ferroptosis tumors had higher TMB levels (*p*=0.028; **Figure 7E**). The ferroptosis score and TMB were negatively correlated (*p*=0.019; **Figure 7F**). Further evidence showed that poor prognosis was strongly associated with high TMB and low ferroptosis scores (*p*<0.001; **Figure 7G**). Considering the synergistic effect of TMB and ferroptosis scores on the prognosis, we conducted a hierarchical prognostic analysis. We found that patients with high ferroptosis score and low TMB had a great survival advantage (*p*<0.001; **Figure 7H**). These data indicate that ferroptosis score combined with TMB can further improve the prognosis of patients.

### 3.10 Ferroptosis score, TME features, and response to immunotherapy

**Figure 8:**
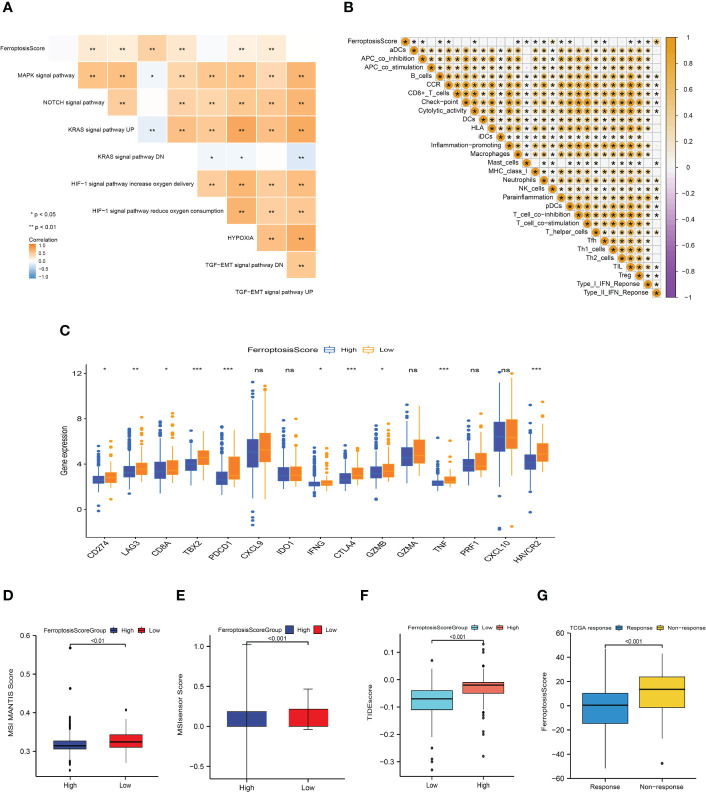
Ferroptosis score correlated with immunotherapy efficacy and response to immunotherapy. **(A, B)** Heat maps of the correlation between ferroptosis score and carcinogenic related signaling pathways **(A)** and immune cell infiltration **(B)**. *p<0.05; **p<0.01; ***p<0.001. **(C)** Box plot depicting the differences between the ferroptosis score groups in the relative expression of checkpoints. ns: not significant; *p<0.05; **p<0.01; ***p<0.001. **(D, E)** Differences between ferroptosis score groups and MSI MANTIS score **(D)** and MSIsensor score **(E)**. **(F)** Box plot depicting the differences between the ferroptosis score groups in the TIDE score. **(G)** Box plot depicting the differences of ferroptosis score between the immunotherapy response groups.

The single sample Gene Set Enrichment Analysis (ssGSEA) results showed that the ferroptosis score was significantly correlated with hypoxia, NOTCH, KRAS, and TGF-EMT signaling pathways (**Figure 8A**). An immune correlation analysis conducted in **Figure 8B** revealed a significant positive relationship between ferroptosis score and NK cells, T helper cells, type-I, and type-II IFN responses, and negatively correlated with immunosuppressive cell Tregs. Based on these findings, it was again confirmed that ferroptosis could affect tumor growth and progression by regulating the tumor microenvironment.

Basic research and clinical trials to exploring the predictive efficacy of immunotherapy biomarkers remain limited. To analyze the immunological response and tolerance to immunotherapy in HCC patients, we chose CD274, CTLA4, LAG3, HAVCR2, IDO1, and PDCD1 as immune checkpoint-related signatures and CD8A, CXCL10, GZMA, CXCL9, GZMB, GZMA, IFNG, PRF1, TBX2, and TNF as immunological activity-related signatures. The majority of immunological checkpoints and immunoreactive-related markers were found to be significantly overexpressed in the group with poor ferroptosis scores (**Figure 8C**).

In the process of DNA replication, the base mismatch loses its repair function and causes accumulation, which causes microsatellite instability (MSI), thus increasing the risk of tumor occurrence. Pabolizumab has been approved for use in MSI-H/dMMR solid tumors. This is also the first drug approved by the Food and Drug Administration (FDA) based on molecular markers rather than tumor tissue sources. Therefore, the changes in MSI-H/dMMR status and related molecules in tumor patients have important implications. We evaluated the MSI MANTIS score and microsatellite instability sensor (MSIsensor) score among the ferroptosis scoring groups (*p*<0.01; **Figures 8D, E**). The MSI MANTIS score has a positive correlation with the probability of MSI-H status (66, 67), and MSIsensor is an effective tool to obtain MSI status from standard tumor normal paired sequence data (68). Not surprisingly, both MSI scores were e higher in the low ferroptosis score group.

The Tumor Immune Dysfunction and Exclusion (TIDE) algorithm was used to evaluate the TCGA-LIHC cohort. The TIDE score of the high ferroptosis group was significantly higher than that of the low ferroptosis group, and the ferroptosis score of the ICIs-response group was significantly lower than that of the non-response group (*p*<0.01; **Figures 8F, G**). Together, this evidence strongly supports the effect of ferroptosis scores in predicting immunotherapy outcomes.

### 3.11 Differences in chemotherapy drug sensitivity between ferroptosis score groups

**Figure 9:**
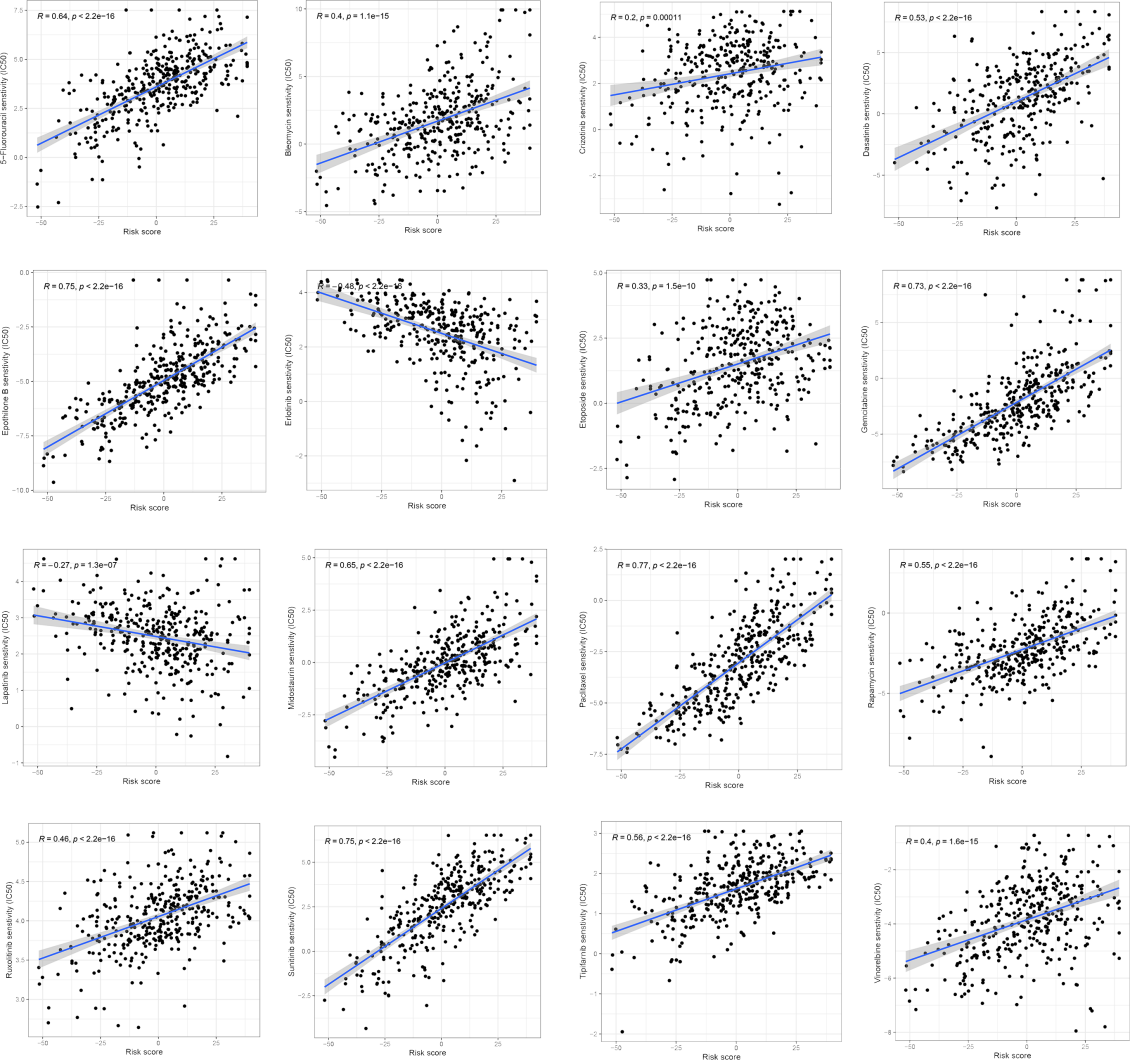
Correlation of ferroptosis scores with chemotherapeutic drug sensitivity.

We examined the relationship between the ferroptosis score and the half-maximum inhibitory concentration (IC_50_) of chemotherapy drugs. Many drugs, including 5-fluorouracil, Dasatinib, Gemcitabine, and a variety of receptor tyrosine kinases (RTK), were significantly associated with the ferroptosis score. Compared with the low ferroptosis score group, the high ferroptosis score group has a higher estimated value of IC_50_ (**Figure 9**). The relationship between ferroptosis score and the semi-maximum inhibitory concentration (IC_50_) of other chemotherapy drugs were shown in the **Supplementary Figure 2**. In conclusion, the high ferroptosis score indicates that HCC patients were more sensitive to these therapeutic drugs.

## 4 Discussion

In recent years, with the tremendous advancement of ICIs monotherapy in the treatment of solid tumors, clinical researchers have conducted extensive research into hepatocellular carcinoma. In 2017, Nivolumab was approved by the FDA to treat Sorafenib treated HCC patients, and became the first immunotherapy drug approved for advanced HCC (2). But the subsequent Checkmate-459 did not meet the primary endpoint, implying that PD-1 inhibitors are effective in hepatocellular carcinoma, but the single-agent efficacy of PD-1 inhibitors still does not fulfill therapeutic needs (69). Subsequently, the combination of PD-L1 inhibitor (Atelizumab) and Bevacizumab (“T+A” scheme for short) in the phase III clinical trial (IMbrave150) for the treatment of advanced hepatocellular carcinoma significantly improved the survival period and quality of life of patients (4, 70, 71). Consequently, with the diversification of systematic treatment schemes for advanced hepatocellular carcinoma, how to accurately select multi-target inhibitors and appropriate immunotherapy schemes has emerged as a hot research topic. Therefore, the key to the treatment of advanced liver cancer in the future is to subdivide patients and find personalized, highly effective, and minimally invasive whole-course treatment strategies to improve long-term survival. Exploring the biomarkers of immunotherapy and molecular targeted therapy based on molecular typing can accurately screen patients who will benefit from immunotherapy and predict the efficacy and prognosis of drugs.

In this study, we identified 32 FRGs that displayed differential expression and a significant correlation with survival in HCC tissues and nearby non-tumor tissues. These FRGs play a vital role in the occurrence, proliferation, metastasis, and even drug resistance of malignant tumors. Then we screened three hub genes (HRAS, SLC7A11, and SLC2A1). We discovered that the high expression of the three hub genes predicted a poor prognosis for patients with liver cancer through the survival analysis of the TCGA cohort, GEO cohort, and the validation cohort of the Affiliated Hospital of Qingdao University. And consistent conclusions were obtained in pan-cancer analysis. Consequently, we identified three molecular subtypes of ferroptosis based on the mRNA expression profiles of FRGs. These three subtypes differ significantly in terms of prognosis, molecular function, immune infiltration microenvironment, and response to immunotherapy. The findings demonstrated a considerable enrichment of NK cell and type II interferon (IFN-γ) response, as well as a particularly pronounced survival advantage for ferroptosis cluster A. It has been proved that NK cells directly kill tumor cells through cytolytic granules and act synergistically with other immune cells through proinflammatory cytokines and chemokines, which is closely related to the prognosis of cancer patients (72–74). At the same time, ferroptosis cluster B was considerably abundant in Tregs and other immunosuppressive cells. Several hypoxia-related and EMT-related signaling pathways (including the TGF-β, MAPK, and KRAS signaling pathways) were also substantially expressed in ferroptosis cluster B. These mechanisms are thought to inhibit T lymphocytes.

The transcriptome differentially expressed genes (DEGs) among different ferroptosis molecular subtypes were particularly enriched for biological processes related to energy metabolism, proliferation, DNA repair, and immune activation. Based on these DEGs, which are considered the characteristic genes related to ferroptosis subtypes, we identified three gene clusters. We found that the ferroptosis-associated genomic patterns almost overlap with the ferroptosis molecular patterns. This implied that there were specific molecular patterns of ferroptosis in HCC. Therefore, a comprehensive assessment of the molecular patterns of ferroptosis is essential to gain insight into HCC. Considering the heterogeneity of HCC, we evaluated each patient ferroptosis molecular patterns by PCA, established ferroptosis scores, and divided HCC patients into groups with high and low ferroptosis scores. Ferroptosis cluster B and gene cluster B had the lowest survival rate and the lowest ferroptosis score, suggesting that a low ferroptosis score may predict unfavorable survival. By combining ferroptosis scores with other independent clinical risk variables, we constructed prognostic multivariate regression models. When compared to other clinical traits, the ferroptosis score contributes the greatest risk factors and has a good prediction efficiency for the outcome of HCC patients. Further investigation into the association between ferroptosis score and clinical characteristics of hepatocellular carcinoma revealed that low ferroptosis score group was significantly related to the features of patients with poor prognosis (such as increased AFP, advanced stage, and poor differentiation). As a result, it was proven that the ferroptosis score was a reliable index for evaluating patient survival.

The prevalence of obesity has reached epidemic proportions and has increased dramatically in recent decades. In addition to causing metabolic and cardiovascular diseases, obesity is also an established risk factor for several gastrointestinal cancers and is strongly associated with pancreatic and liver cancers in particular (75). Therefore, the hepatic molecular mechanisms involving obesity and NAFLD induced hepatocarcinogenesis and potential early markers of HCC are being extensively studied (76). Body mass index (BMI) is a commonly used international standard to measure the degree of obesity and health (77). We investigated the difference in BMI between ferroptosis score groups in patients with hepatocellular carcinoma. The results showed that there was a positive correlation between ferroptosis score and BMI. It has been found that obesity is closely related to the disturbance of iron metabolism, mainly characterized by high ferritin levels (78). Ferroptosis caused by iron accumulation is accompanied by elevated ROS, decreased GSH and inflammatory reactions, insulin resistance and mitochondrial dysfunction, leading to metabolic disorders and the development of obesity (79–81). In terms of immunity, obesity may lead to ferroptosis in Tregs and B1 cells by reducing the levels of NRF2, GPX4 and GCH1 (80, 82). In our previous study, biological processes such as fatty acid and glucose metabolism were also enriched in ferroptosis cluster A. Therefore, it is reasonable to speculate that the occurrence or development mechanism of this subset of HCC patients is related to metabolism.

Several studies have shown that HCC subtypes with poor prognosis may arise from hepatic progenitor cells. The diverse cell origins of HCC may play an important role in the heterogeneous course of HCC. Therefore, we also explored the link between ferroptosis scoring systems and previously developed molecular models. These molecular models focus on the exploration of the tissue origin of HCC and the mechanism between molecular and clinical pathology and clinical behavior. We discovered that the low ferroptosis score group was closely connected to hepatic stem cell origin subtype (CCL subtype, HB16 cluster 2, SOH subtype, HS subtype, and NCIP cluster A) and early recurrence subtype (RS65, SNUR). These subtypes are characterized by a high degree of malignancy, an abundance of hepatic progenitor cell markers (such as cytokeratin 19 and Ep-CAM), a low level of differentiation, increased vascular invasion, and satellite lesions (known risk factors for early recurrence).

Jiang et al. discovered that p53 may inhibit Cys absorption and trigger ferroptosis by preventing SLC7A11 gene expression, thereby inhibiting the growth of tumor cells (83). Woo et al. constructed the p53 signature to evaluate the expression functional status of p53 and found that it was significantly associated with reduced OS. Tumors with low p53 expression were significantly associated with increased copy number instability, increased pathological grading, decreased expression of marker genes in mature hepatocytes, increased risk of tumor recurrence (53, 84, 85). All of these results confirm that the formation of cancers with invasive characteristics is significantly influenced by TP53. In our study, TP53 mutations and p53 signatures were evaluated to determine the functional status and activity of p53. We found that p53 signatures were significantly reduced in the low ferroptosis score group, consistent with the conclusions of Woo et al.

As the main components of tumor microenvironment, immune cells and stromal cells play a crucial role in the regulation of tumor genesis and development. Additionally, immune cell phenotypic and function will be directly impacted by ferroptosis. Our study found that the immune exhaustion subtypes characterized by low ferroptosis score have significant tumor promoting signals (such as activated stroma, T cell exhaustion and immunosuppressive components). Immune dysfunction may be caused by immunosuppressive cells (such as Tregs). The overexpression of immune checkpoint molecules (PD-1, PD-L1, CTLA4, LAG3, and TIM3) in the low ferroptosis score group also indicated T cell exhaustion.

At present, there is no recognized biomarker to accurately predict the efficacy of immunotherapy for HCC. PD-L1 expression, TMB, MSI are the most commonly used indicators to predict the efficacy of ICIs, but their predictive value in HCC lacks the support of high-level clinical evidence (6). The pan-cancer study by Yarchoan et al. demonstrated that patients with high PD-L1 expression and TMB at the same time had the best ICIs efficacy (86). If the PD-L1 level reflects the degree of immune escape from the tumor, TMB represents the immunogenicity of the tumor itself. These are two different dimensions of whether immunotherapy is working. TMB has been found to be inversely associated with survival outcomes in HCC patients, but patients with higher TMB are more likely to respond to checkpoint therapy (87). In contrast to patients with microsatellite instability-low (MSI-L) cancers, those with MSI-high (MSI-H) tumors had a higher response rate to ICIs (88). Therefore, we comprehensively evaluated the immune checkpoint expression, TMB and MSI in HCC patients. We found that there were significant statistical differences among the three indicators in different ferroptosis score groups. Low ferroptosis score was associated with better immunotherapeutic response in all three indicators. Our study also showed that the ferroptosis score combined with TMB could further improve the survival prediction of patients. Additionally, the TIDE score was applied to the TCGA cohort to forecast immunotherapy, which again verified the predictive value of the ferroptosis score for immunotherapy response. According to our analysis of drug sensitivity, the half maximal inhibitory concentration (IC_50_) of several drugs, including 5-fluorouracil, Dasatinib, Gemcitabine, and a variety of receptor tyrosine kinases (RTK), showed a significant positive correlation with the ferroptosis score. The ferroptosis scoring system can stratify patients, screen sensitive patients, and find new methods to overcome the problems related to chemotherapy resistance. These drug sensitivity analyses provide a potential direction for future treatment work.

Overall, our study has a comprehensive exploration of predictive efficacy, clinical characteristics linkage, immune microenvironment, and immunotherapy. We believe that rigorous multifaceted validation analysis will help improve our understanding of this field. However, due to limited time and experimental conditions, there are some unavoidable flaws in our research that cannot be avoided. The specific molecular and biological regulation mechanism of ferroptosis affecting the prognosis of hepatocellular carcinoma has not been verified by experiments. It is hoped that in future studies, we will be able to determine the role of ferroptosis and its related pathways in the development and progression of hepatocellular carcinoma and clarify its signaling mechanisms, which will ultimately help clinicians evaluate the prognosis of hepatocellular carcinoma in order to guide patients to better receive individualized treatment and select appropriate drugs. We hope these studies can provide some valuable clues for future scientific research and clinical practice.

## 5 Conclusions

In this study, we systematically evaluated the ferroptosis molecular patterns in HCC. In order to quantify the ferroptosis status of each patient, we also developed an ferroptosis score. The results showed that ferroptosis score plays a non-negligible role in evaluating the origin of tumor tissue, TME landscape, survival prognosis and predicting the effect of immunotherapy. These results suggested that ferroptosis score might serve as a basis for molecular classification of HCC in order to develop effective targeted therapies and scientifically designed clinical trials.

## Data availability statement

The original contributions presented in the study are included in the article/**Supplementary material**. Further inquiries can be directed to the corresponding author.

## Ethics statement

The studies involving human participants were reviewed and approved by Institutional Review Board of The Affiliated Hospital of Qingdao University. The patients/participants provided their written informed consent to participate in this study.

## Author contributions

Administrative support (JZ), study conception and design (XX), provision of study materials and patients (FY), data collection and assembly (YM), data analysis and interpretation, manuscript writing, final approval of the manuscript (XX, JZ, MJ, XZ). All authors contributed to the article and approved the submitted version.
