# Supplementary material for: Comprehensive characterization of ferroptosis in hepatocellular carcinoma revealing the association with prognosis and tumor immune microenvironment
Source: Front Oncol. 2023 Mar 27;13:1145380. doi: 10.3389/fonc.2023.1145380 (PMC10083400; doi:10.3389/fonc.2023.1145380)
Supplement: Supplementary file 1 [file DataSheet_1.pdf]

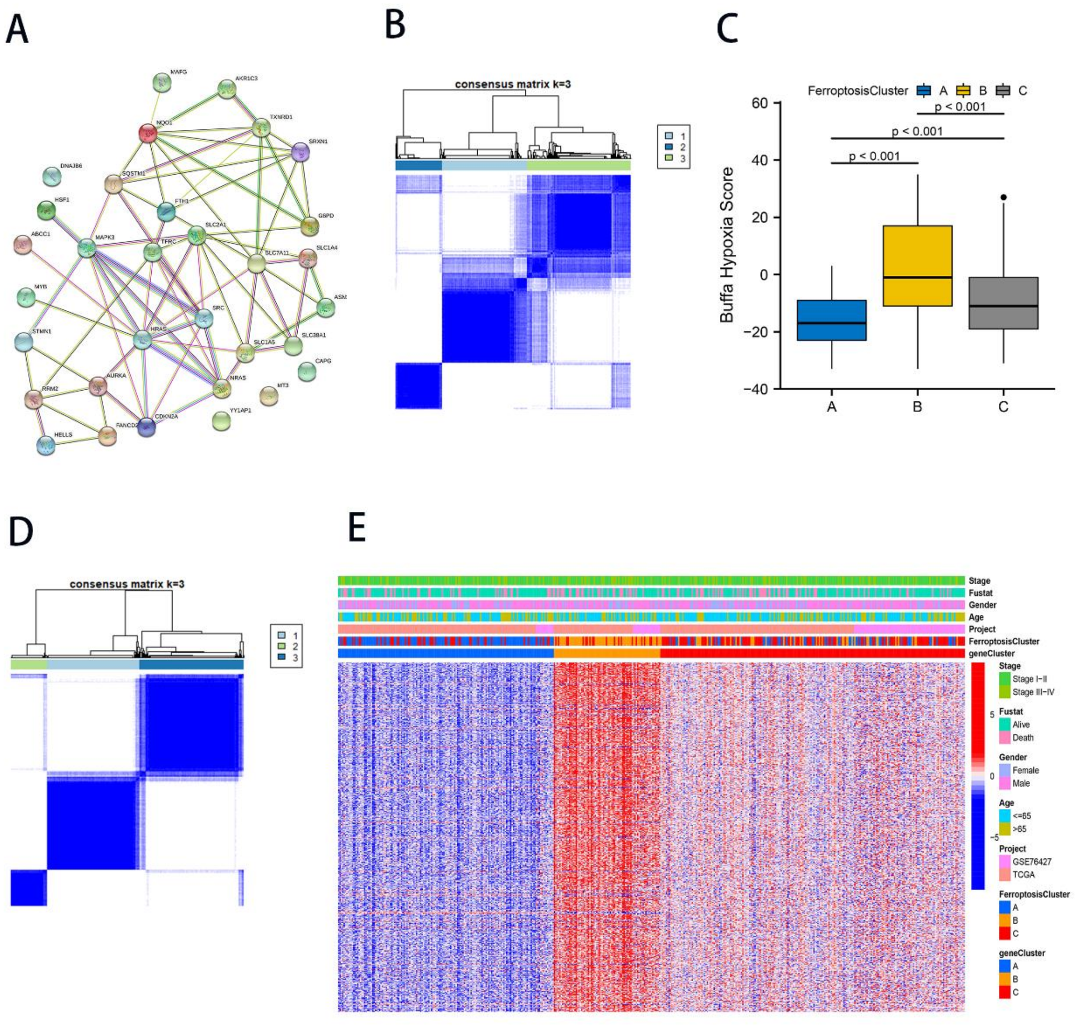

F

Characteristics	Total(N)	HR(95% CI) Univariate analysis	P value Univariate analysis	HR(95% CI) Multivariate analysis	P value Multivariate analysis
Ferroptosis Score group	190		<0.001		
High	160	Reference			
Low	30	2.968 (1.760−5.006)	<0.001	2.757 (1.430−5.315)	0.002
NCIP	190		0.016		
B	124	Reference			
A	66	1.703 (1.103−2.629)	0.016	1.408 (0.769−2.578)	0.268
NCIHS	190		0.991		
HC	163	Reference			
HS	27	1.004 (0.544−1.852)	0.991		
SNUR	190		0.057		
High	70	Reference			
Low	120	0.654 (0.422−1.014)	0.057	0.755 (0.422−1.351)	0.344
HB16	190		0.102		
C1	123	Reference			
C2	67	1.441 (0.930−2.233)	0.102		
RS65	190		0.192		
High	60	Reference			
Low	130	0.742 (0.473−1.161)	0.192		
HIPPO	190		0.511		
AH	146	Reference			
SOH	44	1.177 (0.723−1.918)	0.511		
CC−like	190		0.052		
HCC	136	Reference			
CCL	54	1.576 (0.995−2.495)	0.052	0.675 (0.298−1.526)	0.344

Supplementary Figure 1: (A) The protein interaction network between the FRGs constructed using the Search Tool for the Retrieval of Interacting Genes (STRING) database. **(B)** Unsupervised clustering of FRGs in the TCGA-LIHC and GSE 7642 cohorts. **(C)** Differences of the Buffa Hypoxia Score between the three ferroptosis molecular patterns. **(D)** Unsupervised clustering based on prognostic associated DEGs among ferroptosis clusters in the TCGA-LIHC and GSE 7642 cohorts. **(E)** Heat map illustrating the relationship between ferroptosis clusters

and gene clusters. **(F)** Univariate and Multivariate Cox regression analysis of ferroptosis score groups, CCL-like signature, HB16 signature, NCIP signature, NCIHS signature, RS65 score, SNUR signature, Hippo pathway signature.
