# Supplementary figures and images for: Comprehensive characterization of ferroptosis in hepatocellular carcinoma revealing the association with prognosis and tumor immune microenvironment
Source: Front Oncol. 2023 Mar 27;13:1145380. doi: 10.3389/fonc.2023.1145380 (PMC10083400; doi:10.3389/fonc.2023.1145380)

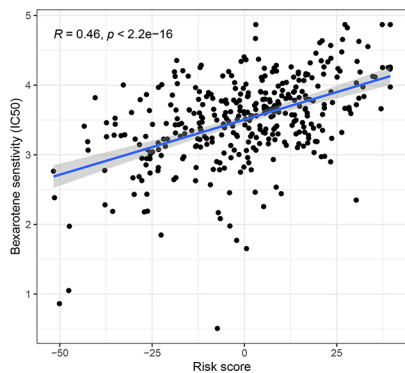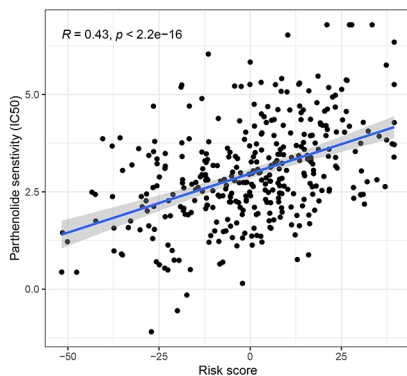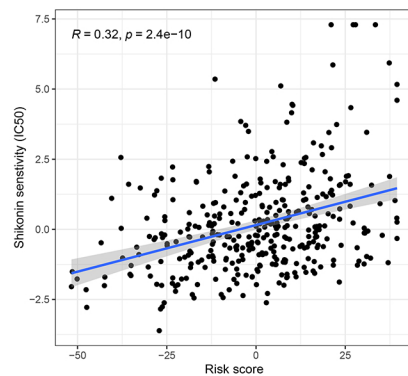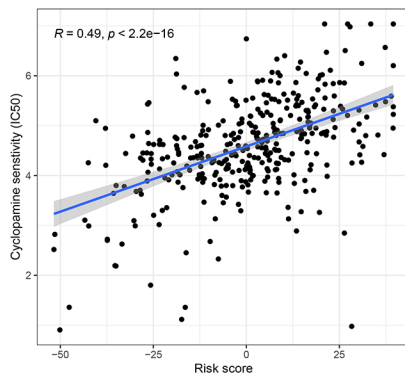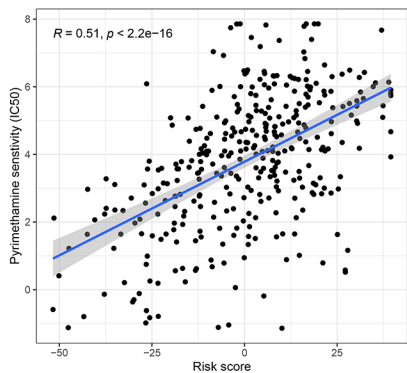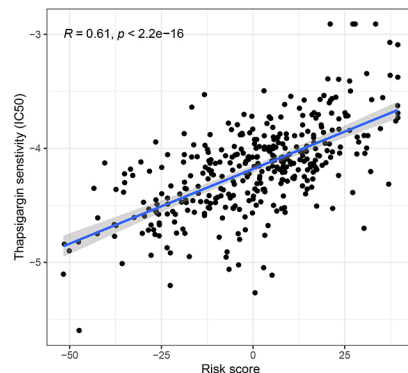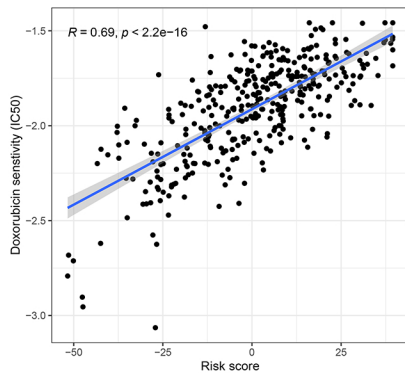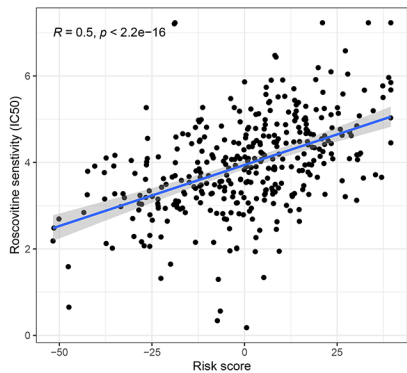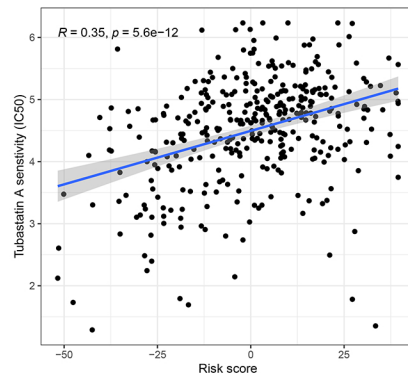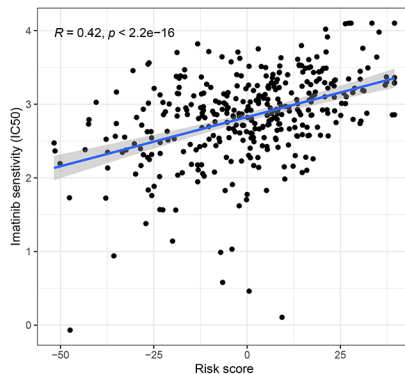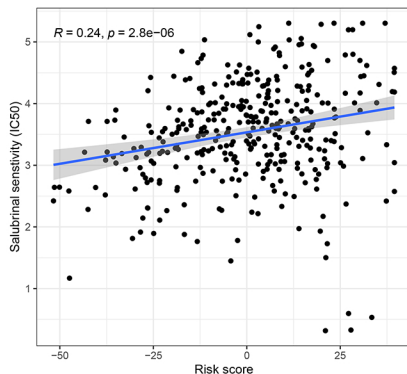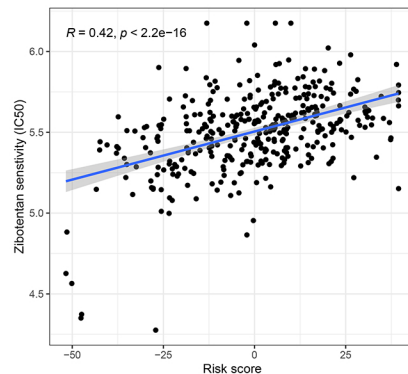

Supplement: Supplementary file 2 [file DataSheet_2.pdf]
