# Supplementary material for: Comprehensive characterization of ferroptosis in hepatocellular carcinoma revealing the association with prognosis and tumor immune microenvironment
Source: Front Oncol. 2023 Mar 27;13:1145380. doi: 10.3389/fonc.2023.1145380 (PMC10083400; doi:10.3389/fonc.2023.1145380)
Supplement: Supplementary file 3 [file Table_1.docx]

**Supplementary Table 1.** **The ferroptosis-related genes obtained from the FerrDb website**

| **Driver** |  | |
| --- | --- | --- |
| Symbol | | Name |
| RPL8 | | Ribosomal protein L8 |
| IREB2 | | Iron response element binding protein 2 |
| ATP5MC3 | | ATP synthase membrane subunit c locus 3 |
| CS | | Citrate synthase |
| EMC2 | | ER membrane protein complex subunit 2 |
| ACSF2 | | Acyl-CoA synthetase family member 2 |
| NOX1 | | Nicotinamide adenine dinucleotide phosphate (NADPH) oxidase (NOX) 1 |
| CYBB | | Cytochrome b-245 beta chain |
| NOX3 | | Nicotinamide adenine dinucleotide phosphate (NADPH) oxidase (NOX) 3 |
| NOX4 | | Nicotinamide adenine dinucleotide phosphate (NADPH) oxidase (NOX) 4 |
| NOX5 | | Nicotinamide adenine dinucleotide phosphate (NADPH) oxidase (NOX) 5 |
| DUOX1 | | Dual oxidase 1 |
| DUOX2 | | Dual oxidase 2 |
| G6PD | | Glucose-6-phosphate dehydrogenase |
| PGD | | Phosphoglycerate dehydrogenase |
| VDAC2 | | Valtage-dependent anion channels 2 |
| PIK3CA | | Phosphatidylinositol-4,5-bisphosphate 3-kinase catalytic subunit alpha |
| FLT3 | | Fms related tyrosine kinase 3 |
| SCP2 | | Sterol carrier protein 2 |
| TP53 | | Tumor protein p53 |
| ACSL4 | | Acyl-CoA synthetase long chain family member 4 |
| LPCAT3 | | Lysophosphatidylcholine acyltransferase 3 |
| NRAS | | NRAS proto-oncogene, GTPase |
| KRAS | | KRAS proto-oncogene, GTPase |
| HRAS | | HRas proto-oncogene, GTPase |
| TF | | Transferrin |
| TFRC | | Transferrin receptor |
| TFR2 | | Transferrin receptor 2 |
| SLC38A1 | | Solute carrier family 38 member 1 |
| SLC1A5 | | Solute carrier family 1 member 5 |
| GLS2 | | Glutaminase 2 |
| GOT1 | | Glutamic-oxaloacetic transaminase 1 |
| CARS1 | | Cysteinyl-tRNA synthetase 1 |
| TP53 | | Tumor protein p53 |
| ALOX5 | | Arachidonate 5-lipoxygenase |
| KEAP1 | | Kelch like ECH associated protein 1 |
| HMOX1 | | Heme oxygenase 1 |
| TP53 | | Tumor protein p53 |
| TP53 | | Tumor protein p53 |
| GLS2 | | Glutaminase 2 |
| ATG5 | | Autophagy related 5 |
| ATG7 | | Autophagy related 7 |
| NCOA4 | | Nuclear receptor coactivator 4 |
| TF | | Transferrin |
| ALOX5 | | Arachidonate 5-lipoxygenase |
| ALOX12 | | Arachidonate 12-lipoxygenase, 12S type |
| ALOX12B | | Arachidonate 12-lipoxygenase, 12R type |
| ALOX15 | | Arachidonate 15-lipoxygenase |
| ALOX15B | | Arachidonate 15-lipoxygenase type B |
| ALOXE3 | | Arachidonate lipoxygenase 3 |
| PHKG2 | | Phosphorylase kinase catalytic subunit gamma 2 |
| TFRC | | Transferrin receptor |
| ACO1 | | Aconitase 1 |
| IREB2 | | iron responsive element binding protein 2 |
| SLC38A1 | | Solute carrier family 38 member 1 |
| GLS2 | | Glutaminase 2 |
| G6PDX | | _NA_ |
| ULK1 | | Unc-51 like autophagy activating kinase 1 |
| ATG3 | | Autophagy related 3 |
| ATG4D | | Autophagy related 4D cysteine peptidase |
| ATG5 | | Autophagy related 5 |
| BECN1 | | Beclin 1 |
| MAP1LC3A | | Microtubule associated protein 1 light chain 3 alpha |
| GABARAPL2 | | GABA type A receptor associated protein like 2 |
| GABARAPL1 | | GABA type A receptor associated protein like 1 |
| ATG16L1 | | Autophagy related 16 like 1 |
| WIPI1 | | WD repeat domain, phosphoinositide interacting 1 |
| WIPI2 | | WD repeat domain, phosphoinositide interacting 2 |
| SNX4 | | Sorting nexin 4 |
| ATG13 | | Autophagy related 13 |
| ULK2 | | Unc-51 like autophagy activating kinase 2 |
| NCOA4 | | Nuclear receptor coactivator 4 |
| ACSL4 | | Acyl-CoA synthetase long chain family member 4 |
| TP53 | | Tumor protein p53 |
| SAT1 | | Spermidine/spermine N1-acetyltransferase 1 |
| ALOX15 | | Arachidonate 15-lipoxygenase |
| ACSL4 | | Acyl-CoA synthetase long chain family member 4 |
| LPCAT3 | | Lysophosphatidylcholine acyltransferase 3 |
| ALOX15 | | Arachidonate 15-lipoxygenase |
| ACSL4 | | Acyl-CoA synthetase long chain family member 4 |
| KEAP1 | | Kelch like ECH associated protein 1 |
| EGFR | | Epidermal growth factor receptor |
| NOX4 | | NADPH oxidase 4 |
| MAPK3 | | Mitogen-activated protein kinase 3 |
| MAPK1 | | Mitogen-activated protein kinase 1 |
| BID | | BH3 interacting domain death agonist |
| ACSL4 | | Acyl-CoA synthetase long chain family member 4 |
| ZEB1 | | Zinc finger E-box binding homeobox 1 |
| KEAP1 | | Kelch like ECH associated protein 1 |
| DPP4 | | Dipeptidyl peptidase 4 |
| ALOX15 | | Arachidonate 15-lipoxygenase |
| ALOX12 | | Arachidonate 12-lipoxygenase, 12S type |
| CDKN2A | | Cyclin dependent kinase inhibitor 2A |
| PEBP1 | | Phosphatidylethanolamine binding protein 1 |
| SOCS1 | | Suppressor of cytokine signaling 1 |
| CDO1 | | Cysteine dioxygenase type 1 |
| MYB | | MYB proto-oncogene, transcription factor |
| HMOX1 | | Heme oxygenase 1 |
| MAPK8 | | Mitogen-activated protein kinase 8 |
| MAPK9 | | Mitogen-activated protein kinase 9 |
| MAPK1 | | Mitogen-activated protein kinase 1 |
| MAPK3 | | Mitogen-activated protein kinase 3 |
| SLC1A5 | | Solute carrier family 1 member 5 |
| CHAC1 | | ChaC glutathione specific gamma-glutamylcyclotransferase 1 |
| MAPK14 | | Mitogen-activated protein kinase 14 |
| LINC00472 | | Long intergenic non-protein coding RNA 472 |
| NOX4 | | NADPH oxidase 4 |
| GOT1 | | Glutamic-oxaloacetic transaminase 1 |
| BECN1 | | Beclin 1 |
| PRKAA2 | | Protein kinase AMP-activated catalytic subunit alpha 2 |
| PRKAA1 | | Protein kinase AMP-activated catalytic subunit alpha 1 |
| ELAVL1 | | ELAV like RNA binding protein 1 |
| BAP1 | | BRCA1 associated protein 1 |
| TP53 | | Tumor protein p53 |
| ABCC1 | | ATP binding cassette subfamily C member 1 |
| ACSL4 | | Acyl-CoA synthetase long chain family member 4 |
| MIR6852 | | microRNA 6852 |
| ACVR1B | | Activin A receptor type 1B |
| TGFBR1 | | Transforming growth factor beta receptor 1 |
| BAP1 | | BRCA1 associated protein 1 |
| EPAS1 | | Endothelial PAS domain protein 1 |
| HILPDA | | Hypoxia inducible lipid droplet associated |
| HIF1A | | Hypoxia inducible factor 1 subunit alpha |
| ALOX12 | | Arachidonate 12-lipoxygenase, 12S type |
| ACSL4 | | Acyl-CoA synthetase long chain family member 4 |
| HMOX1 | | Heme oxygenase 1 |
| IFNG | | Interferon gamma |
| ANO6 | | Anoctamin 6 |
| LPIN1 | | Lipin 1 |
| HMGB1 | | High mobility group box 1 |
| TNFAIP3 | | TNF alpha induced protein 3 |
| TLR4 | | Toll like receptor 4 |
| NOX4 | | NADPH oxidase 4 |
| ATF3 | | Activating transcription factor 3 |
| ATM | | ATM serine/threonine kinase |
| YY1AP1 | | YY1 associated protein 1 |
| EGLN2 | | Egl-9 family hypoxia inducible factor 2 |
| MIOX | | Myo-inositol oxygenase |
| TAZ | | Tafazzin |
| MTDH | | Metadherin |
| IDH1 | | Isocitrate dehydrogenase (NADP(+)) 1 |
| SIRT1 | | Sirtuin 1 |
| TAZ | | Tafazzin |
| BECN1 | | Beclin 1 |
| FBXW7 | | F-box and WD repeat domain containing 7 |
| PANX1 | | Pannexin 1 |
| DNAJB6 | | DnaJ heat shock protein family (Hsp40) member B6 |
| BACH1 | | BTB domain and CNC homolog 1 |
| ACSL4 | | Acyl-CoA synthetase long chain family member 4 |
| LONP1 | | Lon peptidase 1, mitochondrial |
| **Suppressor** | |  |
| Symbol | | Name |
| SLC7A11 | | Solute carrier family 7 member 11 |
| GPX4 | | Glutathione peroxidase 4 |
| AKR1C1 | | Aldo-keto reductase family 1 member C1 |
| AKR1C2 | | Aldo-keto reductase family 1 member C2 |
| AKR1C3 | | Aldo-keto reductase family 1 member C3 |
| GPX4 | | Glutathione peroxidase 4 |
| RB1 | | RB transcriptional corepressor 1 |
| HSPB1 | | Heat shock protein family B (small) member 1 |
| HSF1 | | Heat shock transcription factor 1 |
| SLC7A11 | | Solute carrier family 7 member 11 |
| GPX4 | | Glutathione peroxidase 4 |
| GCLC | | Glutamate-cysteine ligase catalytic subunit |
| SLC7A11 | | Solute carrier family 7 member 11 |
| NFE2L2 | | Nuclear factor, erythroid 2 like 2 |
| SQSTM1 | | Sequestosome 1 |
| NQO1 | | NAD(P)H quinone dehydrogenase 1 |
| HMOX1 | | Heme oxygenase 1 |
| FTH1 | | Ferritin heavy chain 1 |
| MUC1 | | Mucin 1, cell surface associated |
| SLC3A2 | | Solute carrier family 3 member 2 |
| MT1G | | Metallothionein 1G |
| NFE2L2 | | Nuclear factor, erythroid 2 like 2 |
| SLC40A1 | | Solute carrier family 40 member 1 |
| SLC7A11 | | Solute carrier family 7 member 11 |
| GPX4 | | Glutathione peroxidase 4 |
| SLC7A11 | | Solute carrier family 7 member 11 |
| CISD1 | | CDGSH iron sulfur domain 1 |
| SLC7A11 | | Solute carrier family 7 member 11 |
| FANCD2 | | FA complementation group D2 |
| GPX4 | | Glutathione peroxidase 4 |
| NFE2L2 | | Nuclear factor, erythroid 2 like 2 |
| FTMT | | Ferritin mitochondrial |
| HSPA5 | | Heat shock protein family A (Hsp70) member 5 |
| ATF4 | | Activating transcription factor 4 |
| SLC7A11 | | Solute carrier family 7 member 11 |
| GPX4 | | Glutathione peroxidase 4 |
| GPX4 | | Glutathione peroxidase 4 |
| HMOX1 | | Heme oxygenase 1 |
| ATF4 | | Activating transcription factor 4 |
| NFE2L2 | | Nuclear factor, erythroid 2 like 2 |
| TP53 | | Tumor protein p53 |
| SLC7A11 | | Solute carrier family 7 member 11 |
| HELLS | | Helicase, lymphoid specific |
| SCD | | Stearoyl-CoA desaturase |
| FADS2 | | Fatty acid desaturase 2 |
| SRC | | SRC proto-oncogene, non-receptor tyrosine kinase |
| STAT3 | | Signal transducer and activator of transcription 3 |
| NFE2L2 | | Nuclear factor, erythroid 2 like 2 |
| PML | | Promyelocytic leukemia |
| MTOR | | Mechanistic target of rapamycin kinase |
| NFS1 | | NFS1 cysteine desulfurase |
| TP63 | | Tumor protein p63 |
| SLC7A11 | | Solute carrier family 7 member 11 |
| TP53 | | Tumor protein p53 |
| CDKN1A | | Cyclin dependent kinase inhibitor 1A |
| MIR137 | | microRNA 137 |
| SLC40A1 | | Solute carrier family 40 member 1 |
| GPX4 | | Glutathione peroxidase 4 |
| GPX4 | | Glutathione peroxidase 4 |
| ENPP2 | | Ectonucleotide pyrophosphatase/phosphodiesterase 2 |
| VDAC2 | | Voltage dependent anion channel 2 |
| FH | | Fumarate hydratase |
| CISD2 | | CDGSH iron sulfur domain 2 |
| SLC40A1 | | Solute carrier family 40 member 1 |
| MIR9-1 | | microRNA 9-1 |
| MIR9-2 | | microRNA 9-2 |
| MIR9-3 | | microRNA 9-3 |
| CBS | | Cystathionine beta-synthase |
| NFE2L2 | | Nuclear factor, erythroid 2 like 2 |
| SQSTM1 | | Sequestosome 1 |
| GPX4 | | Glutathione peroxidase 4 |
| ISCU | | Iron-sulfur cluster assembly enzyme |
| FTH1 | | Ferritin heavy chain 1 |
| ACSL3 | | Acyl-CoA synthetase long chain family member 3 |
| OTUB1 | | OTU deubiquitinase, ubiquitin aldehyde binding 1 |
| CD44 | | CD44 molecule (Indian blood group) |
| LINC00336 | | Long intergenic non-protein coding RNA 336 |
| STAT3 | | Signal transducer and activator of transcription 3 |
| BRD4 | | Bromodomain containing 4 |
| PRDX6 | | Peroxiredoxin 6 |
| MIR17 | | microRNA 17 |
| SCD | | Stearoyl-CoA desaturase |
| SESN2 | | Sestrin 2 |
| NF2 | | Neurofibromin 2 |
| ARNTL | | Aryl hydrocarbon receptor nuclear translocator like |
| HIF1A | | Hypoxia inducible factor 1 subunit alpha |
| JUN | | Jun proto-oncogene, AP-1 transcription factor subunit |
| CA9 | | Carbonic anhydrase 9 |
| HSPA5 | | Heat shock protein family A (Hsp70) member 5 |
| TMBIM4 | | Transmembrane BAX inhibitor motif containing 4 |
| HSPA5 | | Heat shock protein family A (Hsp70) member 5 |
| PLIN2 | | Perilipin 2 |
| MIR212 | | microRNA 212 |
| Fer1HCH | | Ferritin 1 Heavy Chain Homolog |
| AIFM2 | | Apoptosis inducing factor mitochondria associated 2 |
| AIFM2 | | Apoptosis inducing factor mitochondria associated 2 |
| LAMP2 | | Lysosomal associated membrane protein 2 |
| ZFP36 | | ZFP36 ring finger protein |
| GPX4 | | Glutathione peroxidase 4 |
| PROM2 | | Prominin 2 |
| CHMP5 | | Charged multivesicular body protein 5 |
| CHMP6 | | Charged multivesicular body protein 6 |
| AKR1C1 | | Aldo-keto reductase family 1 member C1 |
| AKR1C2 | | Aldo-keto reductase family 1 member C2 |
| AKR1C3 | | Aldo-keto reductase family 1 member C3 |
| CBS | | Cystathionine beta-synthase |
| NFE2L2 | | Nuclear factor, erythroid 2 like 2 |
| CAV1 | | Caveolin 1 |
| GCH1 | | GTP cyclohydrolase 1 |
| **Marker** | |  |
| Symbol | | Name |
| PTGS2 | | Prostaglandin-endoperoxide synthase 2 |
| DUSP1 | | Dual specificity phosphatase 1 |
| NOS2 | | Nitric oxide synthase 2 |
| NCF2 | | Neutrophil cytosolic factor 2 |
| MT3 | | Metallothionein 3 |
| UBC | | Ubiquitin C |
| ALB | | Albumin |
| TXNRD1 | | Thioredoxin reductase 1 |
| SRXN1 | | Sulfiredoxin 1 |
| GPX2 | | Glutathione peroxidase 2 |
| BNIP3 | | BCL2 interacting protein 3 |
| OXSR1 | | Oxidative stress responsive kinase 1 |
| SELENOS | | Selenoprotein S |
| ANGPTL7 | | Angiopoietin like 7 |
| CHAC1 | | ChaC glutathione specific gamma-glutamylcyclotransferase 1 |
| SLC7A11 | | Solute carrier family 7 member 11 |
| DDIT4 | | DNA damage inducible transcript 4 |
| LOC284561 | | _NA_ |
| ASNS | | Asparagine synthetase (glutamine-hydrolyzing) |
| TSC22D3 | | TSC22 domain family member 3 |
| DDIT3 | | DNA damage inducible transcript 3 |
| JDP2 | | Jun dimerization protein 2 |
| SESN2 | | Sestrin 2 |
| SLC1A4 | | Solute carrier family 1 member 4 |
| PCK2 | | Phosphoenolpyruvate carboxykinase 2, mitochondrial |
| TXNIP | | Thioredoxin interacting protein |
| VLDLR | | Very low density lipoprotein receptor |
| GPT2 | | Glutamic--pyruvic transaminase 2 |
| PSAT1 | | Phosphoserine aminotransferase 1 |
| LURAP1L | | Leucine rich adaptor protein 1 like |
| SLC7A5 | | Solute carrier family 7 member 5 |
| HERPUD1 | | Homocysteine inducible ER protein with ubiquitin like domain 1 |
| XBP1 | | X-box binding protein 1 |
| ATF3 | | Activating transcription factor 3 |
| SLC3A2 | | Solute carrier family 3 member 2 |
| CBS | | Cystathionine beta-synthase |
| ATF4 | | Activating transcription factor 4 |
| ZNF419 | | Zinc finger protein 419 |
| KLHL24 | | Kelch like family member 24 |
| TRIB3 | | Tribbles pseudokinase 3 |
| ZFP69B | | ZFP69 zinc finger protein B |
| ATP6V1G2 | | ATPase H+ transporting V1 subunit G2 |
| VEGFA | | Vascular endothelial growth factor A |
| GDF15 | | Growth differentiation factor 15 |
| TUBE1 | | Tubulin epsilon 1 |
| ARRDC3 | | Arrestin domain containing 3 |
| CEBPG | | CCAAT enhancer binding protein gamma |
| SNORA16A | | Small nucleolar RNA, H/ACA box 16A |
| RGS4 | | Regulator of G protein signaling 4 |
| BLOC1S5-TXNDC5 | | BLOC1S5-TXNDC5 readthrough (NMD candidate) |
| LOC390705 | | _NA_ |
| EIF2S1 | | Eukaryotic translation initiation factor 2 subunit 1 |
| KIM-1 | | Kidney injury molecule-1 |
| IL6 | | Interleukin 6 |
| CXCL2 | | C-X-C motif chemokine ligand 2 |
| RELA | | RELA proto-oncogene, NF-kB subunit |
| HSD17B11 | | Hydroxysteroid 17-beta dehydrogenase 11 |
| AGPAT3 | | 1-acylglycerol-3-phosphate O-acyltransferase 3 |
| SETD1B | | SET domain containing 1B, histone lysine methyltransferase |
| HMOX1 | | Heme oxygenase 1 |
| TF | | Transferrin |
| FTL | | Ferritin light chain |
| RPL8 | | Ribosomal protein L8 |
| ATP5MC3 | | ATP synthase membrane subunit c locus 3 |
| TFRC | | Transferrin receptor |
| MAFG | | MAF bZIP transcription factor G |
| IL33 | | Interleukin 33 |
| FTH1 | | Ferritin heavy chain 1 |
| SLC40A1 | | Solute carrier family 40 member 1 |
| TF | | Transferrin |
| TFRC | | Transferrin receptor |
| FTH1 | | Ferritin heavy chain 1 |
| GPX4 | | Glutathione peroxidase 4 |
| HAMP | | Hepcidin antimicrobial peptide |
| HSPB1 | | Heat shock protein family B (small) member 1 |
| NFE2L2 | | Nuclear factor, erythroid 2 like 2 |
| STEAP3 | | STEAP3 metalloreductase |
| DRD5 | | Dopamine receptor D5 |
| GPX4 | | Glutathione peroxidase 4 |
| DRD4 | | Dopamine receptor D4 |
| MAP3K5 | | Mitogen-activated protein kinase kinase kinase 5 |
| MAPK14 | | Mitogen-activated protein kinase 14 |
| SLC2A1 | | Solute carrier family 2 member 1 |
| SLC2A3 | | Solute carrier family 2 member 3 |
| SLC2A6 | | Solute carrier family 2 member 6 |
| SLC2A8 | | Solute carrier family 2 member 8 |
| SLC2A12 | | Solute carrier family 2 member 12 |
| GLUT13 | | _NA_ |
| SLC2A14 | | Solute carrier family 2 member 14 |
| EIF2AK4 | | Eukaryotic translation initiation factor 2 alpha kinase 4 |
| EIF2S1 | | Eukaryotic translation initiation factor 2 subunit alpha |
| ATF4 | | Activating transcription factor 4 |
| ALOX5 | | Arachidonate 5-lipoxygenase |
| ALOX12 | | Arachidonate 12-lipoxygenase, 12S type |
| ALOX15 | | Arachidonate 15-lipoxygenase |
| ALOX5 | | Arachidonate 5-lipoxygenase |
| ACSF2 | | Acyl-CoA synthetase family member 2 |
| IREB2 | | Iron responsive element binding protein 2 |
| GPX4 | | Glutathione peroxidase 4 |
| HMGB1 | | High mobility group box 1 |
| HMOX1 | | Heme oxygenase 1 |
| NFE2L2 | | Nuclear factor, erythroid 2 like 2 |
| ELAVL1 | | ELAV like RNA binding protein 1 |
| SLC3A2 | | Solute carrier family 3 member 2 |
| SLC7A11 | | Solute carrier family 7 member 11 |
| TFAP2C | | Transcription factor AP-2 gamma |
| SP1 | | Sp1 transcription factor |
| HBA1 | | Hemoglobin subunit alpha 1 |
| NNMT | | Nicotinamide N-methyltransferase |
| PLIN4 | | Perilipin 4 |
| HIC1 | | HIC ZBTB transcriptional repressor 1 |
| STMN1 | | Stathmin 1 |
| RRM2 | | Ribonucleotide reductase regulatory subunit M2 |
| CAPG | | Capping actin protein, gelsolin like |
| HNF4A | | Hepatocyte nuclear factor 4 alpha |
| NGB | | Neuroglobin |
| YWHAE | | Tyrosine 3-monooxygenase/tryptophan 5-monooxygenase activation protein epsilon |
| GABPB1 | | GA binding protein transcription factor subunit beta 1 |
| AURKA | | Aurora kinase A |
| MIR4715 | | microRNA 4715 |
| RIPK1 | | Receptor interacting serine/threonine kinase 1 |
| PRDX1 | | Peroxiredoxin 1 |
| MIR30B | | microRNA 30b |
