# Supplementary material for: Comprehensive characterization of ferroptosis in hepatocellular carcinoma revealing the association with prognosis and tumor immune microenvironment
Source: Front Oncol. 2023 Mar 27;13:1145380. doi: 10.3389/fonc.2023.1145380 (PMC10083400; doi:10.3389/fonc.2023.1145380)
Supplement: Supplementary file 4 [file Table_2.docx]

**A series of gene sets that associated with some biological processes.**

| **Gene Signature** | **Genes** | **Source** |
| --- | --- | --- |
| EMT1 | CLDN3, CLDN7, CLDN4, CDH1, VIM, TWIST1, ZEB1, ZEB2 | PMID: 24520177 |
| EMT2 | AXL, FAP, LOXL2, ROR2, TAGLN, TWIST2, WNT5A | PMID: 26997480 |
| EMT3 | FOXF1, GATA6, SOX9, TWIST1, ZEB1, ZEB2 | PMID: 27321955 |
| WNT target | EFNB3, MYC, TCF12, VEGFA | PMID: 25970248 |
| Pan-F-TBRS | ACTA2, ACTG2, ADAM12, ADAM19, CNN1, COL4A1, CTGF, CTPS1, FAM101B, FSTL3, HSPB1, IGFBP3, PXDC1, SEMA7A, SH3PXD2A, TAGLN, TGFBI, TNS1, TPM1 | PMID: 29443960 |
| Angiogenesis | CDH5, SOX17, SOX18, TEK | PMID: 22553347 |
| TGF-EMT signal pathway _DN | ADORA2B,AGR2,ALDH1A3,ALDH3A2,ALDH5A1,ANK3,AQP3,AREG,ARHGAP29,ATP8B1,BIRC3,C1orf115,C1orf116,CAVIN2,CD9,CDH1,CEACAM6,CEBPD,CFB,CFH,CITED2,CP,CXADR,CYB5A,CYP1B1,DEFB1,DEPTOR,DST,EHF,ELF3,EMP1,EPAS1,EPB41L4B,EPCAM,ERBB3,EREG,ERMP1,ESRP1,FA2H,FGFBP1,FOXA2,GDF15,GLDC,GPRC5C,GRTP1,GSE1,GULP1,HOOK1,HPGD,HS3ST1,IMPA2,INHBB,JAG2,KITLG,KRT15,KRT19,LAMA5,LCN2,LSR,LY6E,MANSC1,MAP7,MBP,METTL7A,MITF,MMP7,MPZL2,MTUS1,MUC1,MYO5C,NR2F2,NUP210,PDK4,PEG10,PKP2,PLAAT3,PLAAT4,PLS1,PPL,PPP1R9A,RAB25,RAB26,RAB38,RBM47,S100P,SCNN1A,SERPINB1,SLC16A7,SLC27A2,SLCO4A1,SLPI,SMPDL3B,SORD,SOX2,SPRY1,SQOR,SULT1A1,SYBU,SYNE2,TBC1D8,TFAP2A,TJP2,TMEM30B,TNFAIP2,TPD52L1,TSPAN1,VAV3, | MSigDB |
| TGF-EMT signal pathway _UP | ACTN1,ADAM12,ADAM19,ADAMTS6,AKT3,ALOX5AP,AMIGO2,ANGPTL4,ANKLE2,AP1S2,APBB2,ARFGAP1,ARHGEF40,ARNTL,BHLHE40,BMP1,BMP2,BMPR2,BPGM,C3orf52,CALD1,CCN2,CD59,CDH11,CDH2,CDK14,CHRNA9,CHST11,COL1A1,COL3A1,COL4A1,COL4A2,COL5A1,COL5A2,COL6A3,COL7A1,CRLF1,CYTH1,DAAM1,DACT1,DHRS2,DIXDC1,DLC1,DOCK4,DSE,DUSP10,ELK3,EML1,EPHB2,ETS2,FAM114A1,FBN1,FERMT2,FHOD3,FN1,FOXD1,FSTL3,GADD45B,GAL,GALNT10,GASK1B,GFPT2,GLIPR1,GNG11,GRB10,GREM1,HMGA2,HMOX1,HRH1,HS3ST3A1,HS3ST3B1,HSF2BP,HTRA1,IGFBP5,IGFBP7,IL11,INHBA,INPP4B,ITGA5,ITGB3,JAG1,JARID2,JUN,JUNB,KCNJ15,KCNMA1,KDELR3,KLF7,LAMC2,LARP6,LBH,LMCD1,LOX,LUM,MAF,MAGED2,MAP1LC3B,MATN3,MBOAT2,MFAP2,MICAL2,MMP1,MMP10,MMP2,MMP9,MN1,MRC2,MYL9,MYO10,NCF2,NEDD9,NKX3-1,NREP,NRIP3,NT5E,NUAK1,PALLD,PDGFA,PDGFC,PDLIM7,PEA15,PID1,PIK3CD,PLAUR,PLEK2,PMEPA1,PODXL,POSTN,PRR5L,PSMD2,PTHLH,PTPN21,PTPRK,PXDC1,RALA,RFTN1,RGS4,RUNX2,SACS,SCG2,SCG5,SEMA3C,SERPINE1,SERPINE2,SIK1,SKIL,SLC22A4,SLC26A2,SLCO2A1,SLN,SMAD7,SMURF2,SNAI2,SPARC,SPDL1,SPHK1,SPOCK1,SRPX,SRRD,STC1,TAGLN,TAGLN2,TBX3,TCF4,TFPI2,TGFB1,TGFB1I1,TGFBI,TGM2,THBS1,TIMP2,TMCC1,TNFAIP6,TNS1,TP53I3,TPM1,TPM4,TPST1,TPST2,TUBA1A,TUBA4A,TUFT1,VCAN,VEGFC,VGLL3,VIM,WNT5A,WNT5B,XYLT1,ZNF365, | MSigDB |
| MAPK signal pathway | AKT2,AKT3,ARRB1,ARRB2,ATF2,ATF4,BDNF,BRAF,CACNA1A,CACNA1B,CACNA1C,CACNA1D,CACNA1E,CACNA1F,CACNA1G,CACNA1H,CACNA1I,CACNA1S,CACNA2D1,CACNA2D2,CACNA2D3,CACNA2D4,CACNB1,CACNB2,CACNB3,CACNB4,CACNG1,CACNG2,CACNG3,CACNG4,CACNG5,CACNG6,CACNG7,CACNG8,CASP3,CD14,CDC25B,CDC42,CHP1,CHP2,CHUK,CRK,CRKL,DAXX,DDIT3,DUSP1,DUSP10,DUSP14,DUSP16,DUSP2,DUSP3,DUSP4,DUSP5,DUSP6,DUSP7,DUSP8,DUSP9,ECSIT,EGF,EGFR,ELK1,ELK4,FAS,FASLG,FGF1,FGF10,FGF11,FGF12,FGF13,FGF14,FGF16,FGF17,FGF18,FGF19,FGF2,FGF20,FGF21,FGF22,FGF23,FGF3,FGF4,FGF5,FGF6,FGF7,FGF8,FGF9,FGFR1,FGFR2,FGFR3,FGFR4,FLNA,FLNB,FLNC,FOS,GADD45A,GADD45B,GADD45G,GNA12,GNG12,GRB2,HRAS,HSPA1A,HSPA1B,HSPA1L,HSPA2,HSPA6,HSPA8,HSPB1,IKBKB,IKBKG,IL1A,IL1B,IL1R1,IL1R2,JMJD7-PLA2G4B,JUN,JUND,KRAS,LAMTOR3,MAP2K1,MAP2K2,MAP2K3,MAP2K4,MAP2K5,MAP2K6,MAP2K7,MAP3K1,MAP3K11,MAP3K12,MAP3K13,MAP3K14,MAP3K2,MAP3K20,MAP3K3,MAP3K4,MAP3K5,MAP3K6,MAP3K7,MAP3K8,MAP4K1,MAP4K2,MAP4K3,MAP4K4,MAPK1,MAPK10,MAPK11,MAPK12,MAPK13,MAPK14,MAPK3,MAPK7,MAPK8,MAPK8IP1,MAPK8IP2,MAPK8IP3,MAPK9,MAPKAPK2,MAPKAPK3,MAPKAPK5,MAPT,MAX,MECOM,MEF2C,MKNK1,MKNK2,MOS,MRAS,MYC,NF1,NFATC2,NFATC4,NFKB1,NFKB2,NGF,NLK,NR4A1,NRAS,NTF3,NTF4,NTRK1,NTRK2,PAK1,PAK2,PDGFA,PDGFB,PDGFRA,PDGFRB,PLA2G10,PLA2G12A,PLA2G12B,PLA2G1B,PLA2G2A,PLA2G2C,PLA2G2D,PLA2G2E,PLA2G2F,PLA2G3,PLA2G4A,PLA2G4B,PLA2G4E,PLA2G5,PLA2G6,PPM1A,PPM1B,PPP3CA,PPP3CB,PPP3CC,PPP3R1,PPP3R2,PPP5C,PRKACA,PRKACB,PRKACG,PRKCA,PRKCB,PRKCG,PRKX,PTPN5,PTPN7,PTPRR,RAC1,RAC2,RAC3,RAF1,RAP1A,RAP1B,RAPGEF2,RASA1,RASA2,RASGRF1,RASGRF2,RASGRP1,RASGRP2,RASGRP3,RASGRP4,RELA,RELB,RPS6KA1,RPS6KA2,RPS6KA3,RPS6KA4,RPS6KA5,RPS6KA6,RRAS,RRAS2,SOS1,SOS2,SRF,STK3,STK4,STMN1,TAB1,TAB2,TAOK1,TAOK2,TAOK3,TGFB1,TGFB2,TGFB3,TGFBR1,TGFBR2,TNF,TNFRSF1A,TP53,TRAF2,TRAF6, | MSigDB |
| HIF-1 signal pathway increase oxygen delivery | EPO,TF,TFRC,VEGF,FLT1,EGF,SERPINE1,ANGPT,TEK,EDN1,NOS2,NOS3,HMDOX1,NPPA TIMP1, | MSigDB |
| HIF-1 signal pathway reduce oxygen consumption | SLC2A1,PDK1,HK,PFKL,ALDOA,ENO1,GAPDH,PGK1,PFKFB3,LDHA,BCL2,CDKN1A,CDKN1B, | MSigDB |
| NOTCH signal pathway | JAG1,NOTCH3,NOTCH2,APH1A,HES1,CCND1,FZD1,PSEN2,FZD7,DTX1,DLL1,FZD5,MAML2,NOTCH1,PSENEN,WNT5A,CUL1,WNT2,DTX4,SAP30,PPARD,KAT2A,HEYL,SKP1,RBX1,TCF7L2,ARRB1,LFNG,PRKCA,DTX2,ST3GAL6,FBXW11, | MSigDB |
| KRAS signal pathway _UP | ANGPTL4,ITGA2,SPRY2,HBEGF,RBP4,HSD11B1,ETV4,GLRX,DUSP6,SCG5,ETV5,ITGB2,AKT2,PPBP,G0S2,GABRA3,IRF8,BIRC3,FGF9,DCBLD2,INHBA,TFPI,TSPAN1,ADAM8,SLPI,PRKG2,MMP11,MMP10,TMEM158,TNFAIP3,PRDM1,GALNT3,ETS1,MMP9,WNT7A,IGFBP3,SPP1,ETV1,CLEC4A,CCND2,TSPAN7,ITGBL1,EMP1,CDADC1,KIF5C,TRIB2,SDCCAG8,PCP4,CFHR2,ALDH1A2,NR0B2,ALDH1A3,AMMECR1,SATB1,GUCY1A1,CSF2,APOD,TOR1AIP2,CMKLR1,TMEM176B,ADGRA2,LAPTM5,CD37,CAB39L,CIDEA,ZNF639,IL1B,GYPC,LY96,FLT4,SPON1,BMP2,PLEK2,IGF2,NR1H4,SNAP25,ACE,PRRX1,C3AR1,TRAF1,TLR8,ID2,TMEM100,PLAUR,GADD45G,CBX8,SCN1B,PTBP2,NAP1L2,AKAP12,PLAT,SCG3,ANO1,IL1RL2,CXCL10,ATG10,YRDC,HDAC9,PEG3,SEMA3B,TNNT2,LIF,CFB,BTC,PPP1R15A,PTPRR,CCL20,ARG1,RETN,KLF4,MMD,PDCD1LG2,H2BC3,HOXD11,TRIB1,F2RL1,ANXA10,TSPAN13,MTMR10,CFH,LAT2,ERO1A,RELN,KCNN4,TMEM176A,MAP4K1,PTGS2,IL33,MAFB,LCP1,NGF,CA2,SERPINA3,RGS16,CTSS,USP12,CPE,SPARCL1,ABCB1,USH1C,CSF2RA,BTBD3,IL2RG,DNMBP,IL10RA,EREG,PRELID3B,EPHB2,FBXO4,CROT,MPZL2,ANKH,CBR4,DOCK2,GPRC5B,RABGAP1L,MALL,STRN,ST6GAL1,PIGR,VWA5A,PSMB8,F13A1,NRP1,SOX9,JUP,ADGRL4,ZNF277,EPB41L3,PCSK1N,FUCA1,PLVAP,ADAM17,AVL9,ADAMDEC1,HKDC1,MAP7,IL7R,RBM4,BPGM,ENG,GFPT2,PLAU,GNG11,PTCD2,MAP3K1,CBL,CXCR4,NIN,IKZF1,WDR33,MYCN,FCER1G,PECAM1,CCSER2,SNAP91,EVI5,TNFRSF1B,GPNMB,TPH1, | MSigDB |
| KRAS signal pathway _DN | CDH16,SPTBN2,FGFR3,NOS1,PDE6B,SIDT1,NTF3,SCN10A,TAS2R4,DTNB,HTR1D,MAST3,SLC6A3,CLDN8,TGM1,PCDHB1,THRB,YPEL1,RYR2,GDNF,CAMK1D,AMBN,LFNG,SERPINA10,ALOX12B,CALCB,FGGY,SPRR3,ATP6V1B1,EDN1,UPK3B,GAMT,PRODH,RYR1,GPR19,SLC29A3,EDN2,GPRC5C,C5,ZC2HC1C,EDAR,KCNMB1,TENT5C,STAG3,KRT4,SLC25A23,INSL5,GP1BA,KMT2D,ABCG4,MYH7,BRDT,PTGFR,KCNN1,SELENOP,IFI44L,PROP1,LGALS7,BMPR1B,PDK2,DCC,SNCB,COL2A1,UGT2B17,NPY4R,DLK2,WNT16,SLC6A14,ITGB1BP2,SLC12A3,YBX2,CKM,CPB1,MAGIX,ARHGDIG,CALML5,KRT1,MTHFR,ABCB11,MYOT,CELSR2,KLK7,THNSL2,CHRNG,NR4A2,CD40LG,KRT13,GP2,CD207,CCR8,ZBTB16,PRKN,CPA2,MEFV,CCNA1,SLC38A3,KLK8,CYP39A1,KCNQ2,SLC30A3,CHST2,ITIH3,EGF,MSH5,TEX15,CLPS,ENTPD7,CLSTN3,CYP11B2,CLDN16,HSD11B2,COQ8A,HNF1A,FGF22,TFCP2L1,OXT,KCND1,MACROH2A2,NRIP2,RGS11,CPEB3,TG,PTPRJ,NUDT11,FSHB,IDUA,TNNI3,GTF3C5,TFF2,SSTR4,COPZ2,PDCD1,SLC16A7,KCNE2,MFSD6,KLHDC8A,CNTFR,IL5,NPHS1,SCGB1A1,CCDC106,PAX3,VPREB1,TSHB,CACNA1F,AKR1B10,P2RX6,KRT15,PAX4,BTG2,GPR3,GRID2,TCL1A,PLAG1,PKP1,IRS4,IL12B,EPHA5,SOX10,SNN,CACNG1,SPHK2,IGFBP2,CAPN9,TCF7L1,TGFB2,SMPX,LYPD3,PNMT,SYNPO,MX1,IFNG,NR6A1,ACTC1,RSAD2,ADRA2C,BARD1,HTR1B,FGF16,TENM2,CDKAL1,SHOX2,SGK1,RIBC2,SKIL,NGB,ASB7,MYO15A,SLC5A5,KRT5,ZNF112,TFAP2B,VPS50,CD80,ATP4A,ARPP21,SERPINB2,TLX1,EFHD1,P2RY4, | MSigDB |
| HYPOXIA | PGK1,PDK1,GBE1,PFKL,ALDOA,ENO2,PGM1,NDRG1,HK2,ALDOC,GPI,MXI1,SLC2A1,P4HA1,ADM,P4HA2,ENO1,PFKP,AK4,FAM162A,PFKFB3,VEGFA,BNIP3L,TPI1,ERO1A,KDM3A,CCNG2,LDHA,GYS1,GAPDH,BHLHE40,ANGPTL4,JUN,SERPINE1,LOX,GCK,PPFIA4,MAFF,DDIT4,SLC2A3,IGFBP3,NFIL3,FOS,RBPJ,HK1,CITED2,ISG20,GALK1,WSB1,PYGM,STC1,ZNF292,BTG1,PLIN2,CSRP2,VLDLR,JMJD6,EXT1,F3,PDK3,ANKZF1,UGP2,ALDOB,STC2,ERRFI1,ENO3,PNRC1,HMOX1,PGF,GAPDHS,CHST2,TMEM45A,BCAN,ATF3,CAV1,AMPD3,GPC3,NDST1,IRS2,SAP30,GAA,SDC4,STBD1,IER3,PKLR,IGFBP1,PLAUR,CAVIN3,CCN5,LARGE1,NOCT,S100A4,RRAGD,ZFP36,EGFR,EDN2,IDS,CDKN1A,RORA,DUSP1,MIF,PPP1R3C,DPYSL4,KDELR3,DTNA,ADORA2B,HS3ST1,CAVIN1,NR3C1,KLF6,GPC4,CCN1,TNFAIP3,CA12,HEXA,BGN,PPP1R15A,PGM2,PIM1,PRDX5,NAGK,CDKN1B,BRS3,TKTL1,MT1E,ATP7A,MT2A,SDC3,TIPARP,PKP1,ANXA2,PGAM2,DDIT3,PRKCA,SLC37A4,CXCR4,EFNA3,CP,KLF7,CCN2,CHST3,TPD52,LXN,B4GALNT2,PPARGC1A,BCL2,GCNT2,HAS1,KLHL24,SCARB1,SLC25A1,SDC2,CASP6,VHL,FOXO3,PDGFB,B3GALT6,SLC2A5,SRPX,EFNA1,GLRX,ACKR3,PAM,TGFBI,DCN,SIAH2,PLAC8,FBP1,TPST2,PHKG1,MYH9,CDKN1C,GRHPR,PCK1,INHA,HSPA5,NDST2,NEDD4L,TPBG,XPNPEP1,IL6,SLC6A6,MAP3K1,LDHC,AKAP12,TES,KIF5A,LALBA,COL5A1,GPC1,HDLBP,ILVBL,NCAN,TGM2,ETS1,HOXB9,SELENBP1,FOSL2,SULT2B1,TGFB3, | MSigDB |
