# Supplementary material for: Comprehensive characterization of ferroptosis in hepatocellular carcinoma revealing the association with prognosis and tumor immune microenvironment
Source: Front Oncol. 2023 Mar 27;13:1145380. doi: 10.3389/fonc.2023.1145380 (PMC10083400; doi:10.3389/fonc.2023.1145380)
Supplement: Supplementary file 5 [file Table_3.docx]

**Supplementary Table 3. The FRGs differentially expressed between HCC tumor tissues and adjacent non-tumor tissues.**

| **Gene** | **conMean** | **treatMean** | | **logFC** | ***p*-value** | **FDR** |
| --- | --- | --- | --- | --- | --- | --- |
| ABCC1 | 0.704233 | 2.385749 | 1.760317 | | 3.35E-06 | 5.37E-06 |
| ACSL4 | 7.308549 | 46.23404 | 2.661299 | | 4.01E-12 | 1.09E-11 |
| AIFM2 | 3.531127 | 9.476027 | 1.424154 | | 1.59E-20 | 1.06E-19 |
| AKR1C1 | 27.57769 | 64.30788 | 1.221494 | | 0.000414 | 0.00056 |
| AKR1C2 | 16.50084 | 50.18181 | 1.604625 | | 9.04E-06 | 1.40E-05 |
| AKR1C3 | 17.81735 | 83.52622 | 2.228946 | | 3.69E-23 | 3.97E-22 |
| ALB | 34415.03 | 14064.25 | -1.29101 | | 3.65E-21 | 2.61E-20 |
| ALOX12 | 0.109919 | 0.275428 | 1.325232 | | 2.53E-12 | 7.06E-12 |
| ALOX15 | 0.022268 | 0.09503 | 2.093416 | | 1.74E-05 | 2.59E-05 |
| ALOX15B | 0.144208 | 1.822449 | 3.659658 | | 1.13E-06 | 1.85E-06 |
| ASNS | 0.501513 | 2.576478 | 2.361042 | | 2.48E-09 | 5.13E-09 |
| ATF3 | 20.75892 | 8.910782 | -1.22011 | | 1.28E-11 | 3.19E-11 |
| AURKA | 0.65871 | 6.887842 | 3.386336 | | 1.38E-27 | 9.87E-26 |
| BAP1 | 7.306535 | 15.19413 | 1.056255 | | 4.79E-23 | 4.91E-22 |
| CAPG | 3.531945 | 13.21648 | 1.903803 | | 4.24E-11 | 9.90E-11 |
| CAV1 | 4.250302 | 8.710903 | 1.035257 | | 7.38E-09 | 1.42E-08 |
| CDKN2A | 0.163454 | 4.102349 | 4.649491 | | 5.33E-25 | 1.27E-23 |
| CS | 5.387151 | 13.82082 | 1.359248 | | 4.34E-22 | 3.59E-21 |
| CXCL2 | 45.4813 | 22.56241 | -1.01135 | | 7.93E-12 | 2.02E-11 |
| DDIT3 | 9.119286 | 25.98337 | 1.510596 | | 4.88E-17 | 2.14E-16 |
| DDIT4 | 14.58734 | 30.42806 | 1.060686 | | 0.000105 | 0.000148 |
| DNAJB6 | 1.463437 | 3.294735 | 1.170801 | | 7.66E-26 | 3.30E-24 |
| DRD4 | 0.155725 | 1.07755 | 2.790687 | | 2.40E-14 | 7.82E-14 |
| DUOX1 | 0.057506 | 0.495322 | 3.106583 | | 1.29E-19 | 7.50E-19 |
| DUSP1 | 210.105 | 101.2582 | -1.05307 | | 1.30E-13 | 3.92E-13 |
| EGLN2 | 1.604616 | 3.795616 | 1.242106 | | 4.81E-20 | 2.87E-19 |
| EMC2 | 4.257881 | 9.93568 | 1.222483 | | 1.21E-23 | 1.57E-22 |
| ENPP2 | 4.194335 | 9.034277 | 1.106967 | | 0.000138 | 0.000191 |
| FANCD2 | 0.131842 | 1.016911 | 2.947314 | | 8.60E-25 | 1.85E-23 |
| FTH1 | 139.2599 | 286.5932 | 1.041224 | | 2.14E-16 | 8.52E-16 |
| FTL | 6043.54 | 12161.39 | 1.008843 | | 1.12E-06 | 1.85E-06 |
| G6PD | 1.309854 | 13.60109 | 3.376244 | | 1.64E-24 | 2.94E-23 |
| GLS2 | 4.839845 | 1.746799 | -1.47025 | | 1.05E-15 | 4.03E-15 |
| HAMP | 147.3988 | 16.00969 | -3.20271 | | 2.04E-24 | 3.37E-23 |
| HELLS | 0.094417 | 0.861944 | 3.190477 | | 1.24E-23 | 1.57E-22 |
| HRAS | 4.550491 | 13.581 | 1.577496 | | 2.60E-24 | 3.99E-23 |
| HSF1 | 6.499934 | 17.7509 | 1.449395 | | 1.06E-24 | 2.07E-23 |
| HSPA5 | 107.2622 | 231.7453 | 1.111398 | | 2.26E-20 | 1.43E-19 |
| HSPB1 | 71.85465 | 338.7734 | 2.237167 | | 2.41E-23 | 2.88E-22 |
| IL6 | 0.87141 | 0.320479 | -1.44312 | | 5.26E-05 | 7.59E-05 |
| MAFG | 1.052752 | 4.16475 | 1.984064 | | 1.82E-22 | 1.57E-21 |
| MAPK3 | 3.6693 | 9.50854 | 1.373719 | | 1.90E-25 | 5.84E-24 |
| MIOX | 0.025345 | 0.72848 | 4.845138 | | 1.89E-11 | 4.61E-11 |
| MT1G | 1211.45 | 316.4162 | -1.93684 | | 1.02E-22 | 9.15E-22 |
| MT3 | 0.017036 | 0.506866 | 4.894922 | | 0.000902 | 0.001192 |
| MYB | 0.046822 | 0.133735 | 1.514108 | | 9.70E-08 | 1.72E-07 |
| NF2 | 1.560253 | 3.969521 | 1.347185 | | 5.66E-24 | 8.11E-23 |
| NNMT | 463.0697 | 141.0111 | -1.71542 | | 3.70E-17 | 1.69E-16 |
| NOS2 | 0.09801 | 0.402883 | 2.039357 | | 1.12E-12 | 3.25E-12 |
| NOX1 | 0.14691 | 0.416211 | 1.502383 | | 1.90E-14 | 6.29E-14 |
| NOX4 | 0.021796 | 0.243084 | 3.479342 | | 6.48E-28 | 6.96E-26 |
| NOX5 | 0.002949 | 0.012785 | 2.11624 | | 1.48E-05 | 2.23E-05 |
| NQO1 | 1.488043 | 58.07197 | 5.286353 | | 2.31E-15 | 8.70E-15 |
| NRAS | 5.744314 | 11.87471 | 1.047685 | | 1.22E-16 | 5.14E-16 |
| PML | 1.497679 | 3.140859 | 1.068431 | | 1.44E-17 | 6.89E-17 |
| PRKAA2 | 0.274825 | 1.62999 | 2.568275 | | 2.90E-10 | 6.43E-10 |
| PTGS2 | 0.687924 | 0.210178 | -1.71063 | | 1.41E-16 | 5.84E-16 |
| RPL8 | 212.7883 | 705.9692 | 1.730186 | | 1.37E-20 | 9.49E-20 |
| RRM2 | 0.472896 | 6.368129 | 3.751273 | | 1.02E-25 | 3.67E-24 |
| SLC1A4 | 1.890788 | 4.998092 | 1.40239 | | 5.71E-15 | 2.08E-14 |
| SLC1A5 | 2.734427 | 8.812906 | 1.688379 | | 0.004382 | 0.005477 |
| SLC2A1 | 0.677907 | 2.538538 | 1.90484 | | 3.98E-05 | 5.78E-05 |
| SLC2A14 | 0.046052 | 0.175056 | 1.926482 | | 0.005202 | 0.006465 |
| SLC2A6 | 0.915939 | 3.118189 | 1.767384 | | 8.11E-08 | 1.45E-07 |
| SLC38A1 | 1.202439 | 4.371047 | 1.862015 | | 1.54E-05 | 2.30E-05 |
| SLC7A11 | 0.028521 | 0.89129 | 4.965815 | | 1.46E-19 | 8.24E-19 |
| SQSTM1 | 29.97898 | 100.0057 | 1.738059 | | 1.78E-16 | 7.20E-16 |
| SRC | 1.483577 | 5.377916 | 1.857968 | | 3.57E-10 | 7.84E-10 |
| SRXN1 | 0.36255 | 1.488197 | 2.037313 | | 1.06E-14 | 3.68E-14 |
| STEAP3 | 47.64738 | 21.8358 | -1.1257 | | 5.14E-19 | 2.70E-18 |
| STMN1 | 1.914111 | 13.5351 | 2.821959 | | 6.39E-27 | 3.44E-25 |
| TAZ | 1.72875 | 5.525063 | 1.676262 | | 2.53E-28 | 5.44E-26 |
| TFRC | 3.665255 | 10.97904 | 1.582766 | | 3.59E-16 | 1.40E-15 |
| TP63 | 0.0325 | 0.122199 | 1.910729 | | 0.002289 | 0.00293 |
| TUBE1 | 3.062087 | 1.327053 | -1.20629 | | 1.63E-20 | 1.06E-19 |
| TXNRD1 | 6.767976 | 28.0904 | 2.053281 | | 4.30E-17 | 1.92E-16 |
| YY1AP1 | 3.838282 | 9.093437 | 1.244365 | | 2.87E-23 | 3.25E-22 |
| ZFP36 | 161.625 | 57.26163 | -1.49701 | | 2.15E-17 | 1.00E-16 |
| ZNF419 | 0.288444 | 0.644296 | 1.159432 | | 2.67E-12 | 7.35E-12 |
|  | | | | | | |
