# Supplementary material for: Comprehensive characterization of ferroptosis in hepatocellular carcinoma revealing the association with prognosis and tumor immune microenvironment
Source: Front Oncol. 2023 Mar 27;13:1145380. doi: 10.3389/fonc.2023.1145380 (PMC10083400; doi:10.3389/fonc.2023.1145380)
Supplement: Supplementary file 6 [file Table_4.docx]

**Supplementary Table 4. The FRGs significantly associated with OS in the Univariate Cox regression analysis.**

| **ID** | **HR** | **HR.95L** | **HR.95H** | ***p*-value** |
| --- | --- | --- | --- | --- |
| ABCC1 | 1.372053 | 1.15562 | 1.629021 | 0.000305 |
| ACSL3 | 1.46127 | 1.148071 | 1.859913 | 0.002057 |
| AKR1C3 | 1.278458 | 1.079392 | 1.514238 | 0.004446 |
| ASNS | 1.30639 | 1.113515 | 1.532673 | 0.001041 |
| ATG13 | 2.072106 | 1.339776 | 3.204734 | 0.001058 |
| ATG16L1 | 1.878052 | 1.182262 | 2.983331 | 0.007607 |
| ATG3 | 2.333713 | 1.530145 | 3.559281 | 8.32E-05 |
| ATG5 | 1.834277 | 1.22314 | 2.750765 | 0.003344 |
| ATG7 | 2.90066 | 1.675243 | 5.02245 | 0.000144 |
| AURKA | 1.321369 | 1.120713 | 1.557952 | 0.000913 |
| CA9 | 1.126153 | 1.042185 | 1.216886 | 0.002655 |
| CAPG | 1.206924 | 1.072293 | 1.358458 | 0.001829 |
| CARS1 | 1.684547 | 1.243 | 2.282943 | 0.000772 |
| CBS | 0.655265 | 0.47684 | 0.900453 | 0.009146 |
| CDKN2A | 1.246458 | 1.075825 | 1.444154 | 0.003357 |
| CDO1 | 0.879197 | 0.805885 | 0.959179 | 0.003754 |
| CISD1 | 1.633011 | 1.197967 | 2.226042 | 0.001918 |
| DNAJB6 | 2.031451 | 1.311832 | 3.145824 | 0.001491 |
| EIF2S1 | 2.460089 | 1.6053 | 3.770034 | 3.58E-05 |
| ELAVL1 | 1.755461 | 1.152225 | 2.674515 | 0.008804 |
| FANCD2 | 1.711206 | 1.292658 | 2.265275 | 0.000174 |
| FTH1 | 1.427981 | 1.141166 | 1.786881 | 0.001844 |
| G6PD | 1.415361 | 1.264493 | 1.584229 | 1.54E-09 |
| GABARAPL1 | 0.790549 | 0.67094 | 0.931481 | 0.004985 |
| HELLS | 1.553531 | 1.195395 | 2.018962 | 0.000985 |
| HIF1A | 1.24797 | 1.062438 | 1.465901 | 0.006986 |
| HILPDA | 1.434723 | 1.239503 | 1.660689 | 1.32E-06 |
| HMOX1 | 1.220242 | 1.065107 | 1.397974 | 0.004116 |
| HRAS | 1.362717 | 1.114051 | 1.666886 | 0.002607 |
| HSF1 | 1.378606 | 1.093262 | 1.738424 | 0.006657 |
| LONP1 | 1.56748 | 1.11702 | 2.199597 | 0.009318 |
| MAFG | 1.759724 | 1.38389 | 2.237627 | 4.02E-06 |
| MAPK3 | 1.573969 | 1.179387 | 2.100565 | 0.002067 |
| MIR9-1 | 3956764 | 108.4194 | 1.44E+11 | 0.004593 |
| MT3 | 1.425206 | 1.163413 | 1.745908 | 0.000622 |
| MYB | 3.097942 | 1.616386 | 5.937469 | 0.000658 |
| NCF2 | 1.31351 | 1.127422 | 1.530313 | 0.000468 |
| NQO1 | 1.102656 | 1.035891 | 1.173725 | 0.002166 |
| NRAS | 1.801248 | 1.378857 | 2.353033 | 1.59E-05 |
| OTUB1 | 1.726051 | 1.223381 | 2.435261 | 0.001884 |
| PGD | 1.501653 | 1.246315 | 1.809304 | 1.91E-05 |
| PRDX1 | 1.658709 | 1.302795 | 2.111857 | 4.02E-05 |
| RRM2 | 1.417725 | 1.206445 | 1.666006 | 2.24E-05 |
| SLC1A4 | 1.45641 | 1.171388 | 1.810783 | 0.000715 |
| SLC1A5 | 1.363718 | 1.215114 | 1.530495 | 1.37E-07 |
| SLC2A1 | 1.532459 | 1.313132 | 1.788419 | 6.07E-08 |
| SLC38A1 | 1.333319 | 1.161364 | 1.530734 | 4.44E-05 |
| SLC7A11 | 1.465883 | 1.232433 | 1.743554 | 1.55E-05 |
| SQSTM1 | 1.380827 | 1.174768 | 1.623029 | 9.10E-05 |
| SRC | 1.244985 | 1.05725 | 1.466057 | 0.008601 |
| SRXN1 | 1.696777 | 1.343932 | 2.142261 | 8.78E-06 |
| STMN1 | 1.425395 | 1.21978 | 1.665669 | 8.21E-06 |
| TFRC | 1.302033 | 1.074892 | 1.577173 | 0.006969 |
| TRIB3 | 1.220094 | 1.049945 | 1.417818 | 0.009432 |
| TXNRD1 | 1.36106 | 1.16981 | 1.583576 | 6.61E-05 |
| VDAC2 | 1.661318 | 1.208285 | 2.284212 | 0.001781 |
| VEGFA | 1.365072 | 1.09825 | 1.69672 | 0.005039 |
| YY1AP1 | 1.635911 | 1.198577 | 2.232818 | 0.001927 |
|  | | | | |
