# Supplementary material for: Comprehensive characterization of ferroptosis in hepatocellular carcinoma revealing the association with prognosis and tumor immune microenvironment
Source: Front Oncol. 2023 Mar 27;13:1145380. doi: 10.3389/fonc.2023.1145380 (PMC10083400; doi:10.3389/fonc.2023.1145380)
Supplement: Supplementary file 7 [file Table_5.docx]

**The relationship between HRAS expression and clinical features in the validation cohort in our hospital**

| **Characteristic** | **High** | **Low** | ***P-*value** |
| --- | --- | --- | --- |
| *n* | 41 | 28 |  |
| **Gender**, *n* (%) |  |  | 0.748 |
| female | 6 (8.7%) | 5 (7.2%) |  |
| male | 35 (50.7%) | 23 (33.3%) |  |
| **Stage**, *n* (%) |  |  | 0.550 |
| stage I | 20 (29%) | 10 (14.5%) |  |
| stage II | 10 (14.5%) | 8 (11.6%) |  |
| stage III | 11 (15.9%) | 10 (14.5%) |  |
| **Age**, median (IQR) | 55 (45, 63) | 57.5 (46.75, 62) | 0.956 |
| **OS** (months), median (IQR) | 19.7 (13.6, 31.4) | 27.6 (22.6, 40.38) | 0.025 |

IQR: interquartile range; OS: overall survival; The OS did not satisfy the normal distribution (*p*<0.05), using the Wilcoxon rank sum test.

**The relationship between SLC2A1 expression and clinical features in the validation cohort in our hospital**

| **Characteristic** | **High** | **Low** | ***P-*value** |
| --- | --- | --- | --- |
| *n* | 32 | 37 |  |
| **Gender**, *n* (%) |  |  | 0.793 |
| female | 6 (8.7%) | 5 (7.2%) |  |
| male | 26 (37.7%) | 32 (46.4%) |  |
| **Stage**, *n* (%) |  |  | 0.473 |
| stage I | 12 (17.4%) | 18 (26.1%) |  |
| stage II | 8 (11.6%) | 10 (14.5%) |  |
| stage III | 12 (17.4%) | 9 (13%) |  |
| **Age**, median (IQR) | 59.5 (47, 64) | 54 (45, 60) | 0.119 |
| **OS** (months), median (IQR) | 23.65 (12.82, 29.1) | 27.4 (19.2, 39.1) | 0.014 |

IQR: interquartile range; OS: overall survival; The OS did not satisfy the normal distribution (*p*<0.05), using the Wilcoxon rank sum test.

**The relationship between SLC7A11 expression and clinical features in the validation cohort in our hospital**

| **Characteristic** | **High** | **Low** | ***P-*value** |
| --- | --- | --- | --- |
| *n* | 38 | 31 |  |
| **Gender**, *n* (%) |  |  | 1.000 |
| female | 6 (8.7%) | 5 (7.2%) |  |
| male | 32 (46.4%) | 26 (37.7%) |  |
| **Stage**, *n* (%) |  |  | 0.215 |
| stage I | 17 (24.6%) | 13 (18.8%) |  |
| stage II | 7 (10.1%) | 11 (15.9%) |  |
| stage III | 14 (20.3%) | 7 (10.1%) |  |
| **Age**, median (IQR) | 55 (44.5, 62.75) | 56 (47, 62.5) | 0.880 |
| **OS** (months), mean ± SD | 22.83 ± 11.24 | 31.53 ± 15.96 | 0.010 |

IQR: interquartile range; OS: overall survival; The OS was normal distribution (*p*<0.05) and satisfied homogeneity of variance (*p*<0.05), using T test.

**The differences in IHC staining scores of hub genes between tumors and adjacent normal tissues (Wilcoxon rank sum test).**

| 组别 | 组别I | 组别J | 统计量 | 差值(J-I) | 置信区间(95%CI) | p值 |
| --- | --- | --- | --- | --- | --- | --- |
| HRAS | Tumor | Adjacent | 2671.5 | -6 | -3.9999 | 4.90E-15 |
| SLC2A1 | Tumor | Adjacent | 2803.5 | -6 | -3 | 5.06E-18 |
| SLC7A11 | Tumor | Adjacent | 2809.5 | -7 | -1.9999 | 3.17E-18 |
