# Supplementary material for: Comprehensive characterization of ferroptosis in hepatocellular carcinoma revealing the association with prognosis and tumor immune microenvironment
Source: Front Oncol. 2023 Mar 27;13:1145380. doi: 10.3389/fonc.2023.1145380 (PMC10083400; doi:10.3389/fonc.2023.1145380)
Supplement: Supplementary file 8 [file Table_6.docx]

**Relationship between ferroptosis score groups and clinical features in TCGA**

| **Characteristics** | **High** | **Low** | **P value** |
| --- | --- | --- | --- |
| n | 307 | 46 |  |
| **Age, median (IQR)** | 61 (52, 69) | 58.5 (48, 66) | 0.070 |
| **Child pugh classification grade, n (%)** |  |  | 0.790 |
| B | 20 (8.7%) | 1 (0.4%) |  |
| A | 190 (82.6%) | 18 (7.8%) |  |
| C | 1 (0.4%) | 0 (0%) |  |
| **Stage, n (%)** |  |  | 0.005 |
| Stage III | 65 (19.6%) | 19 (5.7%) |  |
| Stage I | 152 (45.8%) | 11 (3.3%) |  |
| Stage II | 68 (20.5%) | 12 (3.6%) |  |
| Stage IV | 4 (1.2%) | 1 (0.3%) |  |
| **Grade, n (%)** |  |  | 0.016 |
| G1 | 50 (14.4%) | 2 (0.6%) |  |
| G3 | 94 (27%) | 24 (6.9%) |  |
| G2 | 149 (42.8%) | 18 (5.2%) |  |
| G4 | 9 (2.6%) | 2 (0.6%) |  |
| **Sex, n (%)** |  |  | 0.707 |
| Male | 205 (58.1%) | 32 (9.1%) |  |
| Female | 102 (28.9%) | 14 (4%) |  |
| **BMI, median (IQR)** | 24.984 (21.86, 29.329) | 22.797 (20.832, 25.768) | 0.011 |

IQR: interquartile range; OS: overall survival; BMI: Body Mass Index

Child-Pugh level does not meet the theoretical frequency > 5 or total sample size > 40, Fisher's exact test was used. All levels of Stage, Grade, and Gender meet the theoretical frequency >5 and total sample size > 40, the chi-square test was used. Age, OS, and BMI not satisfying the normal distribution (*p* < 0.05), using the Wilcoxon rank sum test.

**Logistic regression analyzing the relationship between ferroptosis score groups and clinical features, taking the high ferroptosis score group as the reference.**

| **Characteristics** | **Total(N)** | **Univariate analysis** | |  | **Multivariate analysis** | |
| --- | --- | --- | --- | --- | --- | --- |
|  |  | **Odds Ratio (95% CI)** | **P value** |  | **Odds Ratio (95% CI)** | **P value** |
| Age | 353 | 0.983 (0.960 - 1.005) | 0.130 |  |  |  |
| Child-pugh classification | 230 |  |  |  |  |  |
| B | 21 | Reference |  |  |  |  |
| A | 208 | 1.895 (-0.171 - 3.960) | 0.544 |  |  |  |
| C | 1 | 0.000 (-2852.580 - 2852.580) | 0.993 |  |  |  |
| **Stage** | 332 |  |  |  |  |  |
| Stage III | 84 | Reference |  |  | Reference |  |
| Stage I | 163 | 0.248 (-0.550 - 1.045) | < 0.001 |  | 0.321 (-0.510 - 1.153) | 0.007 |
| Stage II | 80 | 0.604 (-0.195 - 1.402) | 0.216 |  | 0.833 (-0.006 - 1.672) | 0.670 |
| Stage IV | 5 | 0.855 (-1.395 - 3.105) | 0.892 |  | 0.774 (-1.498 - 3.047) | 0.825 |
| **Sex** | 353 |  |  |  |  |  |
| Male | 237 | Reference |  |  |  |  |
| Female | 116 | 0.879 (0.208 - 1.551) | 0.707 |  |  |  |
| **BMI** | 321 | 0.924 (0.856 - 0.992) | 0.022 |  | 0.935 (0.866 - 1.003) | 0.054 |

**Relationship between ferroptosis score groups and clinical features in GEO**

| **Characteristic** | **High** | **Low** | ***P-*value** |
| --- | --- | --- | --- |
| *n* | 103 | 11 |  |
| **Gender**, *n* (%) |  |  | 1.000 |
| female | 19 (16.7%) | 2 (1.8%) |  |
| male | 84 (73.7%) | 9 (7.9%) |  |
| **Stage**, *n* (%) |  |  | 0.186 |
| Stage I | 52 (45.6%) | 3 (2.6%) |  |
| Stage II | 30 (26.3%) | 5 (4.4%) |  |
| Stage III | 19 (16.7%) | 2 (1.8%) |  |
| Stage IV | 2 (1.8%) | 1 (0.9%) |  |
| **OS** (months), median (IQR) | 15.24 (5.58, 38.88) | 6.6 (5.22, 16.74) | 0.104 |
| **Age**, median (IQR) | 64 (55, 73) | 65 (56, 71) | 0.777 |

IQR: interquartile range; OS: overall survival

Gender and Stage does not meet the theoretical frequency > 5 or total sample size > 40, Fisher's exact test was used. Age and OS not satisfying the normal distribution (*p* < 0.05), using the Wilcoxon rank sum test.

**Relationship between ferroptosis score groups and clinical features in ICGC**

| **Characteristic** | **High** | **Low** | ***P-*value** |
| --- | --- | --- | --- |
| *n* | 170 | 61 |  |
| **Gender**, *n* (%) |  |  | 0.418 |
| female | 42 (18.2%) | 19 (8.2%) |  |
| male | 128 (55.4%) | 42 (18.2%) |  |
| **Age**, median (IQR) | 69 (62, 74.75) | 67 (62, 73) | 0.385 |
| **OS** (months), median (IQR) | 29.59 (19.73, 38.47) | 17.75 (10.85, 24.66) | < 0.001 |

IQR: interquartile range; OS: overall survival

Gender meet the theoretical frequency > 5 and total sample size > 40, the chi-square test was used. Age and OS not satisfying the normal distribution (*p* < 0.05), using the Wilcoxon rank sum test.

**Relationship between ferroptosis score groups and clinical features in TCGA subtypes**

| **Characteristic** | **High** | **Low** | ***P-*value** |
| --- | --- | --- | --- |
| *n* | 160 | 30 |  |
| **NCIP**, *n* (%) |  |  | < 0.001 |
| A | 39 (20.5%) | 27 (14.2%) |  |
| B | 121 (63.7%) | 3 (1.6%) |  |
| **NCIHS**, *n* (%) |  |  | < 0.001 |
| HC | 147 (77.4%) | 16 (8.4%) |  |
| HS | 13 (6.8%) | 14 (7.4%) |  |
| **SNUR**, *n* (%) |  |  | < 0.001 |
| High | 46 (24.2%) | 24 (12.6%) |  |
| Low | 114 (60%) | 6 (3.2%) |  |
| **HB16**, *n* (%) |  |  | < 0.001 |
| C1 | 121 (63.7%) | 2 (1.1%) |  |
| C2 | 39 (20.5%) | 28 (14.7%) |  |
| **RS65**, *n* (%) |  |  | < 0.001 |
| High | 37 (19.5%) | 23 (12.1%) |  |
| Low | 123 (64.7%) | 7 (3.7%) |  |
| **HIPPO**, *n* (%) |  |  | < 0.001 |
| AH | 132 (69.5%) | 14 (7.4%) |  |
| SOH | 28 (14.7%) | 16 (8.4%) |  |
| **CC-like**, *n* (%) |  |  | < 0.001 |
| CCL | 28 (14.7%) | 26 (13.7%) |  |
| HCC | 132 (69.5%) | 4 (2.1%) |  |
| **Grade**, *n* (%) |  |  | 0.034 |
| G1 | 32 (16.8%) | 2 (1.1%) |  |
| G2 | 81 (42.6%) | 12 (6.3%) |  |
| G3 | 43 (22.6%) | 16 (8.4%) |  |
| unknown | 4 (2.1%) | 0 (0%) |  |
| **AFP** (ng/ml), *n* (%) |  |  | 0.018 |
| <300 | 93 (48.9%) | 10 (5.3%) |  |
| ≥300 | 26 (13.7%) | 5 (2.6%) |  |
| unknown | 41 (21.6%) | 15 (7.9%) |  |
| **OS** (months), median (IQR) | 19.68 (11.77, 29.82) | 9.39 (3.22, 13.67) | < 0.001 |
| **RS65 Score**, median (IQR) | 25.75 (17.37, 37.76) | 68 (44.92, 84.65) | < 0.001 |
| **Age**, median (IQR) | 63 (53, 71) | 57.5 (47.25, 66.25) | 0.055 |

IQR: interquartile range; OS: overall survival

NCIHS, Grade, and AFP level does not meet the theoretical frequency > 5 or total sample size > 40, Fisher's exact test was used. All levels of NCIP, SNUR, HB16, RS65, HIPPO, and CC-like meet the theoretical frequency > 5 and total sample size > 40, the chi-square test was used. RS65 Score, OS, and Age not satisfying the normal distribution (*p* < 0.05), using the Wilcoxon rank sum test.
